# Unlocking the Power of Plant-Derived Natural Products: Therapeutic Benefits for Cognitive Health and Neuropsychiatric Symptoms in Dementia-Related Diseases

**DOI:** 10.3390/plants15111619

**Published:** 2026-05-25

**Authors:** Sachiko Koyama, Linh Pham, Yuka Murakawa, Yoko Ogawa, Kanako Terauchi, Keith Davis

**Affiliations:** 1 Department of Surgery, Indiana University School of Medicine, Indianapolis, IN 46202, USA; 2 Department of Science and Mathematics, Texas A&M University-Central Texas, Killeen, TX 76549, USA; linhpham@tamuct.edu; 3 Faculty of Information Sciences, Hannan University, Matsubara 580-0032, Japan; 4 Independent Researcher, Kyoto 611-0028, Japan; 5 Aromavilla IFA, Department of Integrative Medicine, Osaka ISEN College of Medical Care & Welfare, Matsushita Nursing School, Osaka 573-0075, Japan; aromavilla@snow.ocn.ne.jp; 6 BioScape Innovations, Indianapolis, IN 46278, USA

**Keywords:** dementia, Alzheimer’s disease, phytochemicals, flavonoids, polyphenols, neuroprotection, aromatherapy, neuroinflammation, cognitive decline, natural products

## Abstract

Dementia, including Alzheimer’s disease (AD), represents one of the most pressing public health challenges of the 21st century, affecting more than 55 million individuals worldwide, with projections reaching 139 million by 2050. Current pharmacological treatments offer limited efficacy and significant side effects, driving intense interest in plant-derived natural products as both preventive and therapeutic agents. This review synthesizes preclinical and clinical evidence for key phytochemical classes, including polyphenols, phenolic acids, flavonoids, terpenoids, and alkaloids, in the context of dementia and age-related cognitive decline. Molecular mechanisms are examined in detail, including effects on antioxidant defense and redox homeostasis, suppression of neuroinflammation, and enhancement of synaptic plasticity and neurotransmission. Despite promising preclinical and epidemiological evidence, most clinical trials remain limited in scale and duration and provide mixed results on the efficacy of using phytochemicals for cognitive health. Among the compounds with the most consistent clinical support are the ginkgo diterpene extract EGb 761, saffron carotenoids, curcumin, and rosmarinic acid. A dedicated section addresses the emerging evidence for aromatherapy as a non-pharmacological intervention for behavioral and cognitive symptoms of dementia. Future directions include strategies to improve bioavailability of phytochemicals, the utilization of aromatherapy together with oral supplements, and the need for larger randomized controlled trials using well-characterized and reproducibly manufactured formulations and purified active compounds. Priority areas for future investigation include resolving pharmacokinetic barriers to central nervous system (CNS) delivery, standardizing herbal product composition, and conducting adequately designed clinical trials in well-defined patient populations.

## 1. Introduction

Life expectancy has been extended by approximately 30 years during the last half century in high-income countries [1]. The discoveries of life-saving drugs, the extraordinary advancement in medical treatments, and the improvement in the techniques and awareness related to hygiene in clinical settings have all contributed to saving lives that could not have been saved previously and have extended life expectancies. Another key factor at the individual level is higher levels of prevailing knowledge by the public about healthy lifestyles. The importance of exercise and a healthy diet is well known and may have contributed to the increased number of seniors in relatively good health for their physiological age.

What inevitably come along with extended life expectancy are concerns about cognitive functions, i.e., the possibility of getting dementia, during the later stages of life. Healthy aging includes mental health, including cognitive health, in addition to physical health. How to prevent dementia by maintaining both mental/cognitive and physical health is a significant issue for many people. How to improve the symptoms of dementia is also an important question. Numerous drugs for dementia are currently in clinical trials, and some are approved by the U.S. Food and Drug Administration (FDA) (listed on the website of the Alzheimer’s Association: accessed on 22 February 2026 https://www.alz.org/getmedia/4f4ca289-a2c6-4df9-8cdf-390365bd477e/alzheimers-dementia-fda-approved-treatments-for-alzheimers-ts.pdf). The emergence of these therapies offers hope to patients, though significant obstacles remain, including high costs and the risk of serious adverse events. A detailed summary of currently approved pharmacological treatments for dementia, including their mechanisms of action, routes of administration, and adverse event profiles, is provided in Section 3.2.3, along with an overview of selected clinical trials investigating plant-derived compounds.

How to prevent dementia and how to suppress the symptoms as well as the progress of the disease in our daily lives in a way that is affordable and without adverse events are of broad importance. One such method is through diet [2]. Gut microbiota diversity is positively correlated with antioxidant capacity [2]. Antioxidant, anti-inflammatory, and anti-amyloid properties are found in various phytochemical compounds, suggesting that intake of phytochemical compounds with beneficial bioactive properties could be one of the ways to prevent and/or suppress symptoms related to dementia. While consuming phytochemical-rich vegetables as part of a regular diet is beneficial, achieving therapeutically relevant daily doses of specific compounds through diet alone may not be practical or feasible for all individuals. Other than diet, there are various ways to take phytochemicals. It is possible to take them as supplements, and there are some medicines that have a plant origin. Supplemental and pharmaceutical formulations allow for precise, consistent dosing of non-volatile and low-volatility phytochemicals and are generally the most practical approach to achieving therapeutically relevant daily intake levels.

Another way of getting the beneficial effects of phytochemicals that not many people consider is using essential oils. Many people still consider essential oils as a product for relaxation while enjoying pleasant odors because of the lack of information on the effects of phytochemical compounds with bioactive properties included in the essential oils. In this review, we summarize (i) the neuropathology of dementia (Section 3), (ii) the phytochemical compounds with bioactive properties related to biomarkers of dementia (Section 4), (iii) the studies on the effects of essential oils on cognitive function and biomarkers related to dementia (Section 5), and (iv) the obstacles that need to be overcome to effectively utilize essential oils for patients with dementia (Section 6). A recent review also examined plant-derived natural products in the context of neurodegenerative diseases with organizational similarities to the present work [3]. The present review is distinguished by its broader coverage of phytochemical classes including phytosterols and alkaloids, its dedicated treatment of aromatherapy and essential oils as delivery routes for volatile phytochemicals, and its integration of novel administration strategies including nose-to-brain and transdermal pathways as routes for both essential oil delivery and pharmaceutical development.

## 2. Literature Search Strategy

This narrative review was conducted through systematic searches of PubMed/MEDLINE, Scopus, and Google Scholar. Searches were performed between August 2025 and May 2026 using the following primary search terms and their combinations: dementia, Alzheimer’s disease, mild cognitive impairment, phytochemicals, flavonoids, polyphenols, phenolic acids, terpenoids, alkaloids, phytosterols, essential oils, aromatherapy, neuroprotection, neuroinflammation, amyloid-beta, tau protein, and cognitive decline. Additional targeted searches were conducted for individual compounds and plant species discussed in each section. Priority was given to primary experimental studies, systematic reviews, and meta-analyses published in English in peer-reviewed journals. Clinical trials were prioritized in the treatment sections. Reference lists of relevant reviews were also screened for additional citations.

## 3. Mild Cognitive Impairment (MCI) and Dementia

### 3.1. MCI

This section provides an overview of the clinical spectrum from pre-dementia cognitive impairment to established dementia, followed by a discussion of neuropathological mechanisms, available biomarkers, and current pharmacological management as a foundation for understanding how phytochemicals may contribute to prevention and treatment. Before a person is diagnosed with dementia, there is a transitional stage called MCI. The criteria of MCI seem rather vague, with some studies reporting that normal daily life can be presumed and general cognitive function is normal and not demented, but noticeable objective memory impairment is reported by the person or informant [4], whereas some studies report significant cognitive symptoms interfering with daily activities [5].

Petersen et al. [6] at the Mayo Clinic, Rochester, MN, USA, reported the first criteria to diagnose MCI, providing differentiation from healthy controls and patients with AD [6]. However, according to the Mayo Clinic website (accessed on 6 November 2025; https://www.mayoclinic.org/diseases-conditions/mild-cognitive-impairment/diagnosis-treatment/drc-20354583), there is still no test to diagnose MCI, indicating the difficulties in diagnosing MCI. Diagnosis is mostly conducted based on the information from the individual or from the informant. In a recent paper by Hessen [7], the author compared the criteria from the National Institute of Aging and the Alzheimer’s Association (NIA-AA), American Psychiatric Association (DSM-5), and the World Health Organization (WHO) (International Classification of Diseases 11th Revision (ICD-11)) [7]. The differences in the criteria of these three organizations are very small. They all list that (1) the person has concerns about changes in cognitive functions (subjective cognitive decline) [4,7,8], that (2) there are no problems in daily life and the person functions independently [4,7,8], and that (3) the changes cannot be explained by other reasons such as delirium or other mental/physical disorder [4,7,8]. The NIA-AA criteria list that “impairment is required in one or more of the following cognitive domains: attention, memory, language, visuospatial skills, and executive function” [7]. The DSM-5 criteria by the American Psychiatric Association also add “socially inappropriate behavior” or “personality change,” and the ICD-11 criteria by WHO request results of cognitive tests in the diagnosis [7] and no dementia [4]. Further work in this field would greatly benefit from a uniform definition of MCI that includes multiple quantifiable symptoms as benchmarks.

From a therapeutic standpoint, the MCI stage represents a critical window for intervention where disease modification may be more achievable than in established dementia. Several of the phytochemical compounds discussed in Section 4 have been evaluated specifically in MCI populations or in models representing early-stage cognitive decline. These include curcumin, which demonstrated improved verbal memory in healthy older adults and individuals with subjective cognitive decline [9]; rosmarinic acid, which significantly improved Clinical Dementia Rating-Sum of Boxes (CDR-SB) scores in MCI patients over 4 months [10]; *Centella asiatica* extract, which showed cognitive benefits in cognitively impaired older adults [11]; and the *Ginkgo biloba* extract EGb 761, for which systematic review evidence supports benefits in MCI and early dementia [12]. The applicability of preclinical phytochemical findings to the MCI stage is discussed further in the context of each compound class in Section 4.

### 3.2. Dementia

According to the WHO, 57 million people worldwide have been diagnosed with dementia, and 60% to 70% of the cases were Alzheimer’s disease (AD) (accessed on 15 February 2026 at https://www.who.int/news-room/fact-sheets/detail/dementia#:~:text=In%202021%2C%2057%20million%20people,dependency%20among%20older%20people%20globally). According to the U.S. Centers for Disease Control and Prevention (CDC), 6.7 million people in the United States have AD, which is 60 to 80% of the cases of dementia (accessed on 15 February 2026 at https://www.cdc.gov/alzheimers-dementia/about/index.html). These reports indicate that AD is the most prevalent type of dementia.

The differences between MCI and dementia are explained by the ability of the person to continue daily life independently (Alzheimer’s Foundation of America, accessed on 21 February 2026; https://alzfdn.org/dementia-or-mild-cognitive-impairment and [13]). In the patients who have ‘mild’ dementia, daily activity is affected in that patients can take baths, dress by themselves, and use the bathrooms, but show difficulties in functionally working or other usual activities [14]. In a study in which the Alzheimer’s Disease Assessment Scale-Cognitive Subscale scores of patients with MCI (average age 72.9, *n* = 769, CDR 0.5), with very mild AD (average age 73.1, *n* = 122, CDR 0.5), mild AD (average age 74, *n* = 183, CDR 1.0), and healthy senior controls (average age 70, *n* = 107) were compared, the scores of the MCI group were found to be higher than those of the controls (MCI group 11.3 vs. controls 5.6), but lower than those of patients with very mild or mild AD (very mild 18.0 and mild 25.2) [15]. Interestingly, although the overall (global) Clinical Dementia Rating (CDR) of the MCI patients and patients with very mild AD were both 0.5, i.e., the same, the scores of subcategories such as memory, orientation, and judgment were different between the controls and patients with AD [15]. The hippocampal volume of the MCI patients was also between the volumes of the control group and those of patients with mild AD [15].

#### 3.2.1. Neuropathology of Dementia

Several types of dementia have been classified and recognized by health professionals (accessed on 22 February 2026 https://www.nia.nih.gov/sites/default/files/understanding-types-dementia_0.pdf) (Table 1). AD is the most prevalent and most likely the best-known type of dementia (Table 1), in which extracellular fibrillar amyloid-β (Aβ) peptide accumulates throughout the brain as plaques in early stages [16,17,18,19], followed by the accumulation of tau protein [17,18,19,20]. Synaptic connections are disturbed, causing neurons to weaken and die, causing atrophy. The differences in the types of dementia are based on where these changes take place and/or the types of proteins/peptides involved in causing the malfunction. Memory loss is common in most types of dementia, whereas visual hallucinations and problems in motor functions are typical in Lewy body dementia. Lewy body protein is an abnormal aggregation of a protein called α-synuclein [21]. α-Synuclein is present not only in the nervous system (both central and peripheral) but also in blood cells and non-nervous system tissues. In the nervous system, it is expressed highly in presynaptic nerve terminals and involved in the release and reuptake of neurotransmitters [21,22,23]. When α-synuclein misfolds into a pathological cross-β-sheet structure [21,22,23], it forms an abnormal aggregation called a Lewy body [21,22].

#### 3.2.2. Biomarkers for Detection of Dementia

There have been various biomarkers reported for dementia, especially in AD [17,20,24,25,26,27,28,29]. Table 2 summarizes examples of the biomarkers reported so far. The disorders involved range from amyloidosis, tauopathy (Tau proteins), neurodegeneration (NFL, GFAP), neuroinflammation (YKL-40, sTREM2, GFAP), and synaptic dysfunction (drebrin, neurogranin). The extracellular vesicles (EVs) of dementia patients are known to have an altered EV protein composition and contain elevated levels of Aβ, with the form containing a N-terminal truncation [30,31]. It is important to note, in the case of extracellular vesicles, that although they can spread toxic forms of Aβ to other locations, there is a possibility of utilizing them in EV-based therapies to deliver drugs [31,32]. The summary of AD biomarkers (Table 2) exemplifies the characteristics of the disease: (1) neuroinflammation, (2) abnormal aggregation of peptides and proteins, (3) weakening of neuronal activities due to the accumulation of these peptides and proteins, (4) spreading of these peptides and proteins through EVs, and, ultimately, (5) atrophy of the brain and loss of brain function and motor function.

#### 3.2.3. Drugs and Supplements for Dementia

##### FDA-Approved Drugs

Table 3 summarizes FDA-approved drugs listed on the website of the Alzheimer’s Association (accessed on 26 February 2026, https://www.alz.org/getmedia/4f4ca289-a2c6-4df9-8cdf-390365bd477e/alzheimers-dementia-fda-approved-treatments-for-alzheimers-ts.pdf). Although there are still no medicines that cure AD, there are some FDA-approved drugs for early-stage dementia or MCI. Some of them target the removal of amyloids, for example, Donanemab (Kisunla^TM^; Eli Lilly & Co., Indianapolis, IN, USA) and Lecanemab (LEQEMBI; Eisai Inc., Bunkyo City, Tokyo, Japan, and Biogen Inc., Cambridge, MA, USA), which are anti-amyloid antibodies (Table 3).

Another type of drug targets acetylcholinesterase (AChE), for example, Donepezil (Aricept; Eisai Inc., Bunkyo City, Tokyo, Japan), which is an AChE inhibitor. Acetylcholine is an excitatory neurotransmitter involved in memory and learning, and AChE is an enzyme that breaks down acetylcholine [33]. Studies have shown that AChE is also involved in increasing Aβ production by binding to the enzyme presenilin-1 and increasing the expression of presenilin-1. Presenilin-1 is involved in Aβ production, and, thus, anti-AChE is expected to suppress the degradation of acetylcholine and suppress the production of Aβ [33].

Suvorexant (Belsomra^®^; Merck Rahway, NJ, USA) is a drug to treat insomnia. It is an anti-orexin receptor drug that suppresses the activities of both orexin receptor 1 (OX1R) and orexin receptor 2 (OX2R), and is considered a dual orexin receptor antagonist [34,35]. Insomnia is often reported in patients with dementia, and recent studies have suggested that an increase in orexin is involved in the malfunctioning sleep–wake cycles [36,37], although the results on the altered levels are controversial [37].

Brexpiprazole (Rexulti^®^; Otsuka America Pharmaceutical, Inc., Hayward, CA, USA) is a drug first developed for schizophrenia and as an antidepressant [38]. It is used in the treatment of agitation, which is often observed in patients with dementia [39,40,41]. There are warnings that it increases the risks of fall injuries, hospitalizations, and deaths by cardiocerebrovascular accidents (stroke) in patients using the drug, and its use requires caution [41,42,43].

As can be seen in Table 3, there have been many rather serious adverse events reported in all the drugs listed. Therefore, the development of drugs with fewer adverse events that can enhance patients’ quality of life is needed.

##### Drugs in Clinical Trials and Supplements Using Plant-Derived Natural Products

The website of the National Institute of Aging (NIA) (accessed on 25 February 2026; https://www.nia.nih.gov/research/ongoing-AD-trials) listed 63 ongoing clinical trials in the U.S. related to dementia. Among those, there is an ongoing clinical trial on the effects of plant-origin natural products, focusing on cannabidiol (CBD). There is a recently completed clinical trial using curcumin (Theracumin^®^ containing 90 mg curcumin, Handok Healthcare, Tokyo, Japan), testing its effects on cognitive functions (accessed on 26 February 2026, https://clinicaltrials.gov/study/NCT01383161). A paper published by the group, which summarized the effects on healthy seniors (age between 50 and 90), showed that the primary verbal memory outcome measure (Buschke–Fuld Selective Reminding Test and Consistent Long-Term Retrieval score) was significantly higher in the experimental group (taking Theracumin^®^ twice daily) after 18 months compared to the placebo condition group [9]. Other than curcumin, there are clinical trial studies showing that rosmarinic acid, which is included in, for example, lemon balm (*Melissa officinalis* L.) and sage (*Salvia officinalis*), significantly improved the CDR-SB after it was taken for 4 months [10]. Studies on the mechanisms of action indicate that, in the case of curcumin, it has binding affinity with Aβ and suppresses the aggregation of Aβ into oligomers or fibrils [44]. Aβ is non-toxic as a monomer and turns toxic upon assembly into oligomeric forms ranging from dimers to protofibrils [44]. Curcumin helps with dissociating the fibrils by disrupting β-sheets and prevents aggregation [20,44,45] (see Section 4.2.1). There are various other phytochemical compounds, such as withanolides, ginkgolides, bilobalide, and bacosides, that have been tested for effects on cognitive function and on dementia [46,47,48]. Thus, there is a strong potential in utilizing plant-derived natural products to prevent and improve the symptoms of dementia, and their use warrants increased research. In the next section, the classification of phytochemical compounds in medicinal plants is summarized.

## 4. Phytochemical Compounds in Medicinal Plants

### 4.1. Classification of Phytochemicals

**Figure 1:**
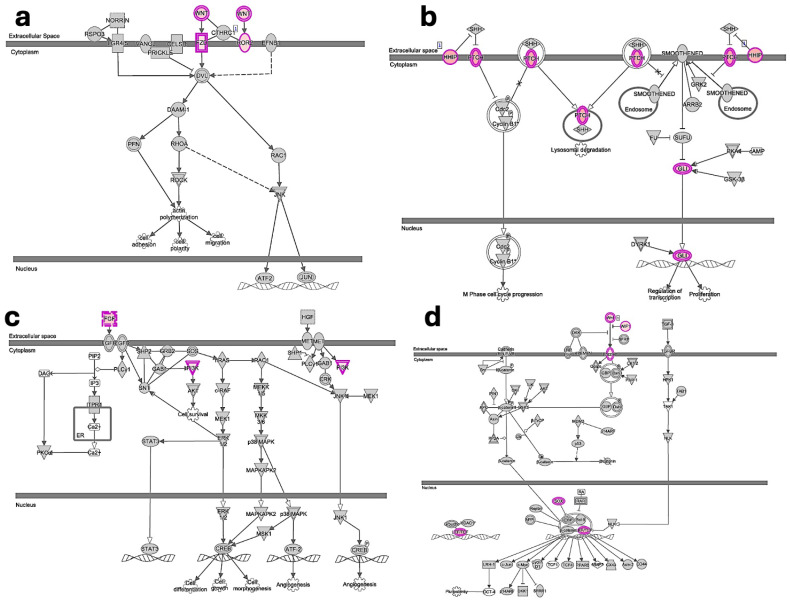
Examples of influence of β-caryophyllene on upregulation of (**a**) Sonic Hedgehog signaling pathways, (**b**) planar cell polarity (PCP) signaling pathway, (**c**) fibroblast growth factor (FGF) signaling pathway, and (**d**) Wnt/beta-catenin signaling pathway in wound healing of mice with experimental skin excision, suggesting its mechanisms of action in enhancing cutaneous re-epithelialization. Pink color indicates genes upregulated in signaling pathway (from Koyama et al. 2019 [50]). (The image is licensed under Creative Commons Attribution License (CC BY 4.0).).

Phytochemicals in medicinal plants are consistently classified into five core secondary metabolite groups: flavonoids, phenolic acids, terpenoids, alkaloids, and phytosterols [49]. The key phytochemicals and the biological pathways linked to each group are detailed in Table 4, and Figure 1 shows examples of the effects of β-caryophyllene from Koyama et al. (2019) [50].

Flavonoids are significant members of the polyphenol family and consist of a general C6–C3–C6 backbone structure, in which C6 and C3 represent a phenyl ring and a heterocyclic pyran ring with an oxygen atom, respectively. They are known for their antioxidant (for example, quercetin), anti-cancer (for example, hesperidin), and anti-inflammatory (for example, kaempferol) activities [49], as well as their ability to protect cardiovascular health (for example, quercetin) [54].

Together with flavonoids, phenolic acids belong to the large polyphenol family and are classified into two subgroups: hydroxybenzoic acids (derived from benzoic acid) and hydroxycinnamic acids (derived from cinnamic acid) [49]. They are recognized for their strong antihypertensive, antidepressant, anti-cancer [55], antidiabetic [56], antioxidant [57], and neuroprotective functions [58].

A diverse class of nitrogen-containing heterocyclic structures derived from amino acids forms the alkaloids group, with prominent representatives such as berberine, caffeine, huperzine A [51], nicotine, morphine, and ephedrine [49]. Their medicinal significance stems from their antispasmodic, antimalarial, antibacterial, and analgesic functions [59,60]. Due to the presence of nitrogen-containing structures, alkaloids often exhibit potent bioactivity through strong interactions with biological receptors and strong radical scavenging abilities, which are critical in the prevention of several degenerative disorders and in inhibiting oxidation reactions [49,61].

Terpenoids, the isoprene-based compounds, constitute one of the largest phytochemical groups, comprising structurally diverse compounds such as ginkgolides, limonene [51], carotenoids, and steroids [49,61]. Their therapeutic values are broad, ranging from anti-inflammatory and sedative activities to immunomodulatory and neuromodulatory effects [62]. They also serve ecological functions such as plant defense and plant-to-plant or plant-to-animal (such as with pollinators) interaction, making them a versatile phytochemical class [57].

Phytosterols, which share structural similarity with cholesterol, are cyclopentaphenanthrene derivatives, with campesterol, β-sitosterol, ergosterol, and stigmasterol representing the major members [49]. They possess a wide range of bioactivities, including anti-cancer [63,64], anti-inflammatory, antioxidant, hepatoprotective [65], and cholesterol-lowering effects [66].

**Figure 2:**
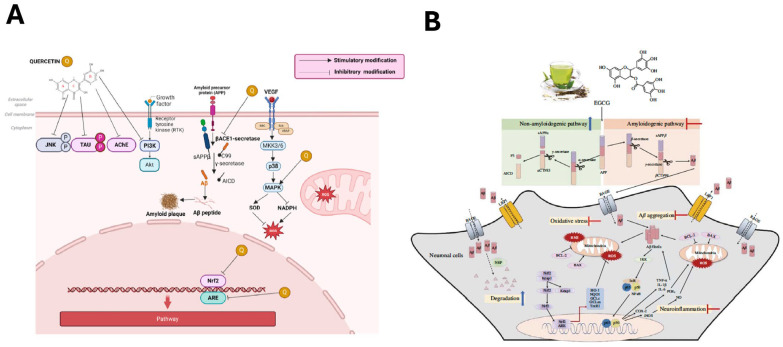
Examples of studies reporting the influence of quercetin (**A**) and EGCG (**B**) on pathways related to dementia. (**A**) is from Frent et al. 2024 [67], and (**B**) is from Youn et al. (2022) [68]. (Both images are licensed under Creative Commons Attribution License (CC BY 4.0).).

Among the compounds cataloged in Table 4, those with the most advanced clinical evidence or compelling translational profiles include quercetin and Epigallocatechin-3-gallate (EGCG) (flavonoids) (Figure 2) for their multi-target anti-amyloid, anti-tau, and neuroprotective activities demonstrated in human studies; curcumin (phenolic component) for its well-documented Aβ-binding and clinical trial data in healthy seniors; EGb 761 (ginkgo terpenoids) for replicated clinical trial evidence in MCI and mild dementia; galantamine (alkaloid) as an FDA-approved acetylcholinesterase (AChE) inhibitor derived from plant sources; and β-sitosterol and 24(S)-saringosterol (phytosterols) for their emerging Liver X receptor (LXR)-mediated cholesterol-regulatory mechanisms in transgenic AD models. These compounds are discussed in detail in the sections that follow.

### 4.2. Biological Pathways of Flavonoids in Dementia-Related Diseases

Flavonoids represent one of the most abundant biologically active plant-derived polyphenols in the human diet [69]. There is significant evidence from epidemiological dietary pattern studies that flavonoid intake is linked with reduced risk of AD and related dementias (ADRD) and reduced loss of cognitive abilities [70,71,72,73,74]. These epidemiological effects have been proposed to be mediated by the neuroprotective effects of flavonoids. Molecular and cellular studies indicate that flavonoid-mediated neuroprotection operates at several levels and involves multiple pathways related to the development of ADRD, the modulation of oxidative stress protection, and signaling pathways that impact the health of neuronal cells.

#### 4.2.1. Inhibition of Aβ Aggregation and Promotion of Clearance

##### Inhibition of Beta-Site Amyloid Cleaving Enzyme 1 (BACE1)

BACE1 is the rate-limiting enzyme of the amyloidogenic pathway, and its inhibition has long been a therapeutic goal in AD. Multiple flavonoids, including quercetin, luteolin, myricetin, and kaempferol, have been shown to inhibit BACE1 activity directly through competitive or non-competitive binding, reducing Aβ generation. Structural modeling identified the catechol moiety on the B ring and the hydroxyl group at position 3 of the C ring as particularly important determinants of BACE1 inhibitory potency. Luteolin is notable for providing a complementary mechanism: in addition to BACE1 inhibition, it upregulates the insulin-degrading enzyme (IDE), a zinc-dependent metalloprotease responsible for Aβ clearance, thereby reducing amyloid burden through parallel routes [75].

##### Disruption of Aβ Aggregation

Several flavonoids interact directly with Aβ monomers and oligomers to destabilize their assembly into toxic fibrils, with EGCG being the most extensively studied compound in this context. Biophysical studies using thioflavin T fluorescence, circular dichroism, and atomic force microscopy have shown that EGCG redirects amyloidogenic polypeptides into unstructured oligomers that are neither toxic nor capable of seeding further aggregation [76]. This occurs by flavonoids forming hydrogen bonds with Aβ peptides and sterically obstructing the β-sheet conformation required for fibril elongation. Quercetin and rutin (the glycoside form of quercetin) similarly exhibit dose-dependent anti-amyloid and fibril-disaggregating activity in vitro and potent antioxidant activity in APP-overexpressing cell lines [77]. Taxifolin has shown efficacy in models of cerebral amyloid angiopathy, preventing both cognitive impairment and vascular amyloid deposition [78]. The catechins, epigallocatechin and epicatechin-3-gallate, block Aβ assembly at sub-micromolar concentrations, adding further structural diversity to the class of flavonoid amyloid-assembly agents [79].

##### Enhancement of Autophagy-Mediated Clearance

Beyond direct anti-aggregation effects, certain flavonoids promote the clearance of existing Aβ and tau deposits through the autophagy-lysosomal pathway. Fisetin, a flavonol found in strawberries and apples, activates the transcription factor EB (TFEB), which drives lysosomal biogenesis and autophagic flux, thereby accelerating degradation of phosphorylated tau and Aβ oligomers [80]. EGCG similarly promotes autophagy through Sirtuin 1 (SIRT1) [81], an additional mechanism that complements its direct anti-aggregation properties.

#### 4.2.2. Suppression of Tau Hyperphosphorylation and Aggregation

Tau hyperphosphorylation in AD is largely mediated by the dysregulated kinases Glycogen synthase kinase-3β (GSK-3β) and cyclin-dependent kinase 5 (CDK5), and multiple flavonoids affect these targets directly or through upstream signaling effects. Quercetin inhibits GSK-3β activity, reducing tau phosphorylation at Ser396 and Thr231 residues, while also activating the Phosphoinositide 3-kinase (PI3K)/Ak strain transforming (Akt) pathway, which phosphorylates and thereby inactivates GSK-3β through a complementary indirect mechanism [82]. Kaempferol modulates GSK-3β through similar Akt-dependent mechanisms, and polyherbal formulations combining kaempferol with quercetin show synergistic reductions in tau pathology in rodent models [83]. Rutin inhibits tau aggregation in cell-free assays and disrupts pre-formed tau fibrils. EGCG prevents tau self-assembly through direct binding that interferes with the intermolecular contacts required for fiber formation [84,85]. Fisetin, through its TFEB-mediated activation of autophagy, also stimulates degradation of phosphorylated tau and has reversed cognitive deficits in P301L tau-expressing mice [80,86].

#### 4.2.3. Attenuation of Neuroinflammation

##### NF-κB Pathway Inhibition

NF-κB is the master transcriptional regulator of pro-inflammatory gene expression in the nervous system, controlling production of Tumor Necrosis Factor alpha (TNF-α), interleukin-1β (IL-1β), IL-6, inducible nitric oxide synthase (iNOS), and cyclooxygenase-2 (COX-2). Multiple flavonoids suppress this pathway in AD-relevant models, with direct evidence linking NF-κB inhibition to cognitive improvement. In the 3 × Tg-AD triple-transgenic mouse model that recapitulates both Aβ and tau pathology, luteolin dose-dependently improved spatial learning and memory in the Morris water maze and was accompanied by significant reductions in TNF-α, IL-1β, IL-6, nitric oxide, COX-2, and iNOS protein levels in brain tissue, along with suppression of astrocyte overactivation [87]. In a complementary Aβ1-42 intracerebroventricular injection model, luteolin directly attenuated phosphorylated NF-κB p65 (Ser536) expression in the frontal cortex and hippocampus, confirming reduced nuclear NF-κB activation in both regions. In vitro studies using a c-Jun NH2-terminal kinase Keap1 (JNK) inhibitor indicate that this effect may be mediated through upstream JNK suppression [88].

The flavonol quercetin suppresses NF-κB activation in microglial cells via a distinct upstream mechanism based on activating SIRT1, causing reduced acetylation of high mobility group box 1 (HMGB1) and restricting its nucleocytoplasmic shuttling, thereby suppressing downstream toll-like receptor 4 (TLR4)/myeloid differentiation primary response 88 (MyD88)/NF-κB signaling [89]. In lipopolysaccharides (LPS)-stimulated BV2 microglia, kaempferol inhibits IκB kinase (IKK) complex activity, preventing Inhibitor of nuclear factor kappa B alpha (IκBα) degradation and NF-κB nuclear translocation, with accompanying reductions in TNF-α, IL-1β, nitric oxide, iNOS, and COX-2 [90]. These microglial pathway studies, while not conducted in ADRD models specifically, support the mechanistic hypothesis of kaempferol and quercetin as NF-κB modulators in the brain inflammatory environment observed in ADRD, and are further supported by parallel findings from hypoxic–ischemic brain injury models in which quercetin’s suppression of TLR4/MyD88/NF-κB signaling in BV2 microglia translated to reduced cerebral infarct volume and rescued cognitive and motor function in neonatal mice [91].

##### Microglial Polarization

Microglia exhibit a range of activation states, ranging from the pro-inflammatory M1-like phenotype that is characterized by cytokine and reactive oxygen species (ROS) release to the anti-inflammatory M2-like cells, which secrete neuroprotective factors and promote Aβ phagocytosis. Several flavonoids shift microglial polarization toward the M2-like state, simultaneously reducing neuroinflammatory activation and enhancing amyloid clearance capacity. Naringenin directly promotes M2 polarization in microglia and upregulates Aβ-degrading enzymes, including neprilysin and insulin-degrading enzyme, suggesting a mechanistic link between polarization state and amyloid clearance [92]. In the amyloid precursor protein (APP)/presenilin1 (PS1) mouse model for AD, naringenin reduces Aβ deposition, suppresses microglial and astrocytic activation, and induces Aβ-degrading enzymes in M2 microglia [93]. Naringenin was also shown to promote microglia M1/M2 polarization to the M2 anti-inflammatory state; this was dependent on reduced c-Jun NH2-terminal kinase (JNK) inactivation, thus implicating mitogen-activated protein kinase (MAPK) signaling as being important [94]. Apigenin reduces M1 microglial activation and decreases cluster of differentiation 68 (CD68) expression and ionized calcium-binding adaptor molecule 1 (Iba-1)+ proliferation in cortical neuron-glia co-cultures exposed to Aβ oligomers, LPS, and IL-1β, with increased brain-derived neurotrophic factor (BDNF) expression and neuronal survival [95]. Similarly, chronic apigenin supplementation reduced hippocampal microgliosis in a chronic neuroinflammatory model relevant to ADRD; however, spatial memory recall impairment was not improved [96]. An additional mechanism through which flavonoids can promote anti-inflammatory microglial polarization in the AD-relevant context is peroxisome proliferator-activated receptor gamma (PPAR-γ) activation in concert with upregulation of triggering receptor expressed on myeloid cell 2 (TREM2) and reduction in Aβ through increased Aβ42 phagocytosis [97].

#### 4.2.4. Antioxidant Defense and Mitochondrial Protection

##### Direct Radical Scavenging

There is strong evidence that flavonoids have the potential to act directly as antioxidant agents. The polyhydroxylated structure of most flavonoids confers potent hydrogen atom transfer and single-electron transfer radical scavenging capacity. The B-ring catechol group (3′,4′-dihydroxylation), the C2=C3 double bond, and the C3 and C5 hydroxyl groups contribute most to this activity. Quercetin and EGCG rank among the most potent natural radical scavengers characterized to date, with measured antioxidant capacities exceeding those of vitamins C and E in several standardized assays [98]. Flavonoids also chelate the redox-active transition metals Fe^2+^ and Cu^2+^ that can catalyze Fenton chemistry and generate highly damaging hydroxyl radicals in proximity to neuronal membranes and deoxyribonucleic acid (DNA) [99].

In AD brains, iron- and Aβ-catalyzed free-radical attack on neuronal membrane polyunsaturated fatty acids generates the toxic aldehyde 4-hydroxynonenal (HNE) at significantly elevated levels beginning at the earliest disease stages, with HNE-protein adducts impairing synaptic and metabolic function [100,101]. Quercetin pretreatment of primary hippocampal neurons in vitro directly attenuates Aβ-induced HNE formation, protein oxidation, and apoptosis in a dose-dependent manner, demonstrating that flavonoid radical scavenging may be able to interrupt this membrane oxidative damage cascade at a biologically relevant point [102].

##### Nuclear Factor Erythroid 2-Related Factor 2 (Nrf2)/Antioxidant Response Element (ARE) Pathway Activation

At the transcriptional level, flavonoids activate the Nrf2/ARE pathway, a master regulator of cellular antioxidant defense. Under basal conditions, Nrf2 is retained in the cytoplasm by its inhibitor Keap1, which targets it for ubiquitin-mediated proteasomal degradation. Flavonoids, including EGCG, genistein, quercetin, and apigenin, promote Nrf2 release and nuclear translocation through mechanisms that include Keap1 protein reduction and upstream PI3K/Akt kinase signaling, activating transcription of heme oxygenase -1 (HO-1), NAD(P)H quinone oxidoreductase 1 (NQO1), superoxide dismutase, catalase, glutathione S-transferase, and glutamate-cysteine ligase [103]. In AD-relevant neuronal models, quercetin activates the kelch-like enoyl-CoA hydratase-associated protein (Keap1)/Nrf2/HO-1 axis in Aβ-challenged PC12 cells and increases cell survival and proliferation [104]. Genistein activates Nrf2 via the PI3K/Akt/Nrf2/Keap1 signaling cascade in primary hippocampal neurons exposed to Aβ25-35, reducing ROS accumulation and neuronal apoptosis [105].

##### Mitochondrial Protection

Flavonoids protect mitochondrial integrity through several converging mechanisms: they prevent mitochondrial membrane depolarization, inhibit cytochrome c release and thereby suppress the intrinsic apoptosis pathway, reduce mitochondrial ROS generation, and enhance ATP synthesis efficiency [106,107]. Quercetin has shown well-characterized mitochondrial protective activity in models of dopaminergic neurodegeneration and AD, including inhibition of caspase-3 activation and Bcl-2-associated X protein (Bax) upregulation [103,108,109].

#### 4.2.5. Modulation of Key Signal Transduction Pathways

##### PI3K/Akt/mTOR Pathway

The PI3K/Akt pathway is a central pro-survival cascade that promotes neuronal survival, inhibits apoptosis, regulates synaptic plasticity, and, through phosphorylation and inactivation of GSK-3β, attenuates tau hyperphosphorylation [110]. Quercetin and several flavonol-enriched plant extracts activate PI3K/Akt signaling, leading to downstream effects on GSK-3β and mTOR [82]. Chronic blueberry supplementation in aged rodents increases hippocampal Akt phosphorylation, activates mechanistic target of rapamycin (mTOR), elevates activity-regulated cytoskeletal-associated protein (Arc/Arg3.1), an activity-regulated cytoskeletal protein important for long-term potentiation consolidation, and improves spatial working memory [111].

##### MAPK/ERK and CREB Signaling

ERK1/2 activation links growth factor receptor signaling to CREB phosphorylation, which drives transcription of synaptic plasticity genes, including BDNF, Arc, and zif268 [112]. Flavonoids and their metabolites interact with MEK1/2 to activate the extracellular signal-regulated kinase (ERK)–cAMP response element-binding protein (CREB) axis and promote memory consolidation. (-)Epicatechin at physiologically relevant nanomolar concentrations activates ERK and stimulates CREB phosphorylation through PI3K-dependent mechanisms in primary cortical neurons, concurrently upregulating glutamate ionotropic receptor AMPA type subunit 2 (GluR2) glutamate receptor subunit expression [113]. Chronic green tea catechin administration activates the protein kinase A (PKA)/CREB pathway and upregulates synaptic plasticity-related proteins, including BDNF, postsynaptic density protein 95 (PSD95), and calcium/calmodulin-dependent protein kinase II (CaMKII), in the hippocampus of senescence-accelerated mouse prone 9 (SAMP8) AD-model mice. This correlated with reductions in Aβ1-42 oligomers and improved spatial memory performance [114]. Flavanols also protect primary neurons and astrocytes from apoptosis by suppressing the pro-apoptotic JNK pathway and its downstream effectors c-jun and caspase-3, driving the signaling pathway toward net neuronal survival [113,115].

##### GSK-3β Inhibition

GSK-3β occupies a pivotal node in AD molecular pathology, phosphorylating tau at multiple disease-relevant epitopes, promoting Aβ production through APP processing modulation, and suppressing neuronal survival signals [116]. Quercetin, kaempferol, myricetin, fisetin, and luteolin reduce tau hyperphosphorylation and enhance cell survival signaling by inhibiting GSK-3β activity through direct adenosine triphosphate (ATP)-competitive kinase inhibition and through PI3K/Akt-mediated phosphorylation of GSK-3β [82,117,118].

##### SIRT1 Activation

SIRT1 is a nicotinamide adenine dinucleotide (NAD)^+^-dependent deacetylase whose expression is significantly reduced in AD brain tissue, with postmortem studies demonstrating a 29–45% decline in SIRT1 mRNA and protein in the parietal cortex of AD patients relative to age-matched controls, and it is correlated with Braak stage and tau accumulation [119]. Through deacetylation of NF-κB p65, SIRT1 suppresses transcriptional activation of pro-inflammatory genes, providing a direct mechanistic link between sirtuin activity and neuroinflammation [120]. SIRT1 also deacetylates and activates peroxisome proliferator-activated receptor gamma coactivator 1-α (PGC-1α), a master transcriptional coactivator of mitochondrial biogenesis, as part of the 5′ adenosine monophosphate-activated protein kinase (AMPK)/SIRT1/PGC-1α axis, which is disrupted in AD. Aβ oligomers reduce SIRT1 and PGC-1α levels in hippocampal neurons, impairing mitochondrial biogenesis [121]. Additional studies showed that SIRT1 is upregulated in AD-related models and that increasing SIRT1 activity by either SIRT1 overexpression or by resveratrol treatment protects against neurodegeneration, reduces hippocampal neuronal loss, and improves learning deficits in the p25 transgenic mouse model [122].

Both quercetin and fisetin activate these SIRT1-dependent pathways, though through partially distinct mechanisms. Quercetin activates SIRT1 to attenuate neuroinflammation via SIRT1/NF-κB-related signaling, including SIRT1/iNOS/NF-κB, and has also been linked to SIRT1-dependent autophagy [123]. Fisetin has been shown to upregulate SIRT1 expression and activate SIRT1-mediated deacetylation in aging brain models, contributing to anti-inflammatory and pro-autophagic effects [124]. Fisetin also promotes autophagic clearance of phosphorylated tau via TFEB and Nrf2 transcription factor activation through mechanistic target of rapamycin complex 1 (mTORC1) inhibition in cortical neurons, an AD-relevant mechanism that operates in parallel to its SIRT1 effects [80].

#### 4.2.6. BDNF Signaling and Neurotrophic Support

BDNF/tropomyosin receptor kinase B (TrkB) signaling is a particularly important target for flavonoid-mediated neuroprotection given the well-documented reduction in BDNF expression in AD [125]. Multiple flavonoid subclasses appear to converge on BDNF/TrkB through overlapping transcriptional and post-translational mechanisms. Upon binding mature BDNF, TrkB activates three principal downstream cascades: MAPK/ERK for differentiation and synaptic plasticity, PI3K/Akt for cell survival, and phospholipase Cγ (PLCγ)/inositol 1,4,5-triphosphate (IP3) for calcium homeostasis and neurotransmission [126,127].

Among the compounds with the most direct relevance to this pathway is 7,8-dihydroxyflavone (7,8-DHF), a naturally occurring flavonoid that functions as a potent TrkB agonist. 7,8-DHF rescues cognitive deficits, synaptic loss, and amyloid burden in multiple AD transgenic mouse models [128,129,130]. Beyond direct TrkB agonism, a wide range of flavonoids upregulate BDNF gene expression: chronic blueberry supplementation elevates hippocampal BDNF levels and CREB phosphorylation; quercetin upregulates BDNF in Aβ-challenged neuronal systems; and kaempferol enhances BDNF-TrkB signaling in hippocampal neurons [131,132,133,134].

#### 4.2.7. Promotion of Adult Neurogenesis

Adult hippocampal neurogenesis involves the generation of new neurons from neural stem cells in the subgranular zone of the dentate gyrus. This process is substantially reduced in AD patients and in transgenic AD models, and this reduction correlates with memory impairment [135]. Several flavonoids promote neurogenesis through BDNF upregulation, PI3K/Akt/mTOR activation, and stimulation of the wingless-related integration site (Wnt)/β-catenin pathway [126]. Quercetin promotes hippocampal neurogenesis in AD-model mice partly through inhibition of endoplasmic integrated stress response signaling that normally suppresses this process [136]. Blueberry-derived anthocyanins and flavanols increase hippocampal neurogenesis in aged rodents, and these histological improvements correlate with enhancements in spatial reference memory [137,138].

#### 4.2.8. Inhibition of AChE and Augmentation of Cholinergic Transmission

A major driver of AD is the progressive degeneration of basal forebrain cholinergic neurons, and this degeneration is thought to be responsible for the episodic memory and attentional deficits associated with AD. In AD, cortical AChE activity is abnormally elevated while choline acetyltransferase (ChAT) is depleted, creating a synaptic ACh deficit that correlates closely with cognitive impairment. As the disease advances, butyrylcholinesterase (BChE) has a major hydrolytic role and both enzymes additionally promote Aβ fibril formation through non-catalytic mechanisms. Thus, there is a dual rationale for their inhibition beyond ACh preservation alone [139,140]. Several flavonoids function as natural inhibitors of AChE and BChE, increasing synaptic acetylcholine availability through mechanisms that overlap pharmacodynamically with approved AD therapies. Among the catechins, EGCG is a competitive inhibitor of both AChE and BChE, and completely reverses the AChE elevation and associated cognitive deficit in a streptozotocin dementia model [141,142]. Flavonoids enhance cholinergic neurotransmission through dual mechanisms: inhibition of ACh degradation and enhancement of ACh synthesis. Quercetin, kaempferol, luteolin, and chrysin inhibit both AChE and BChE activity, slowing synaptic ACh hydrolysis [140,142]. Additionally, quercetin has been shown to upregulate ChAT activity in the hippocampus and prefrontal cortex, thereby directly enhancing ACh synthesis [143].

### 4.3. Biological Pathways of Alkaloids in Dementia-Related Diseases

Several alkaloid families, including indole, isoquinoline, and bisbenzylisoquinoline scaffolds, have been widely investigated for therapeutic actions and show promising neuroprotective effects relevant to dementia therapy [144,145,146]. These compounds engage multi-target mechanisms across enzymes, receptors, transcription factors, mitochondrial dysfunction, autophagy and mitophagy imbalance, and proteostasis pathways, positioning them as strong candidates for addressing the complex pathology of AD and related dementias [51,147].

#### 4.3.1. Neurotransmission, Neurogenesis, and Synaptic Plasticity

A central mechanism of alkaloids in dementia involves cholinergic signaling. Naturally occurring plant-derived alkaloids such as galantamine act as reversible AChE inhibitors and positive allosteric modulators of α7 and α4β2 nicotinic acetylcholine receptors, enhancing cholinergic tone critical for cognition [148]. Several alkaloids also inhibit monoamine oxidases (MAO-A/B), supporting neurotransmitter balance and reducing oxidative burden. This activity is characteristic of β-carboline alkaloids such as harmine and harman, as well as isoquinoline alkaloids like berberine [144,149].

Several isoquinoline, bisbenzylisoquinoneline, and β-carboline alkaloids directly disrupt Aβ fibrillogenesis by binding to Aβ monomers or oligomers, thereby preventing β-sheet formation and destabilizing pre-formed fibrils. Berbamine hydrochloride inhibits the deposition of Aβ plaque by binding to the Aβ fibrils, as demonstrated by molecular docking, chemical kinetics analysis, and atomic force microscopy [150]. P4B is a derivative of papaverine—a benzylisoquinoline alkaloid—with butyrate, which was reported to prevent Aβ aggregation and attenuate oxidative stress in aged APP/PS1 mice [151].

In parallel, alkaloids, such as harmine derivatives, allocryptopine, tetrahydropalmatine, tetrahydroberberine, kopoffines, and ‘dendrobium nobile lindl. alkaloid’ (included in *Dendrobium nobile* Lindl., a traditional Chinese medicinal herb, abbreviated as DNLA), exert significant anti-tau effects. The effects are mediated by (1) suppressing tau phosphorylating kinases such as GSK-3β (harmine derivatives, allocryptopine, tetrahydropalmatine, tetrahydroberberine) [152,153] and CDK5 (kopoffines) [154], and (2) activating pro-survival signaling pathways including PI3K/Akt/GSK-3β (DNLA) [155]. These effects collectively contribute to reducing tau hyperphosphorylation and neurofibrillary tangle formation [156]. By inhibiting dual specificity tyrosine phosphorylation regulated kinase 1A (DYRK1A), harmine has demonstrated its potential in reducing tau hyperphosphorylation, limiting aberrant amyloid precursor protein processing, and enhancing neprilysin-mediated clearance of Aβ [157].

Several studies demonstrated the solid links between alkaloids and adult neurogenesis, including berberine, huperzine A, harmine, galantamine, and vinpocetine. Berberine was suggested to enhance nerve regeneration in hippocampal neurons by modulating the insulin-like growth factor receptor (IGFR)-mediated JNK-Akt signaling pathway [158] and rescue neuronal integrity by suppressing nucleotide-binding oligomerization domain-like receptor family pyrin domain containing 3 (NLRP3) activation, thereby restoring synaptic plasticity and promoting neurogenesis [159]. Ma et al. found that huperzine A promotes hippocampal neurogenesis both in vitro and in vivo by activating the MAPK/ERK signaling pathway [160]. One strong piece of evidence linking the β-carboline alkaloid family to neurogenic pathways is that harmine markedly increases the proliferation of human neural progenitor cells (hNPCs) by inhibiting the dual-specificity tyrosine-phosphorylation-regulated kinase (DYRK1A) [161]. Recently, Jiang et al. showed that galantamine upregulates the insulin-like growth factor 2 (IGF-2) pathway, leading to the elevation of adult neuronal differentiation and neurite outgrowth in neural stem and progenitor cells (NSPCs) [162].

#### 4.3.2. Anti-Inflammatory and Immunomodulatory Pathways

Many alkaloids attenuate neuroinflammation through suppression of the NF-κB and MAPK pathways. Bisbenzylisoquinolines such as liensinine, neferine, and isoliensinine reduce cytokine release and inhibit IκBα phosphorylation in microglia [146,163]. Isoliensinine also diminishes BV2 microglial inflammation by modulating MAPK/NF-κB signaling, reducing oxidative stress and mitochondrial dysfunction [164]. Tetrandrine further downregulates NF-κB/MAPK/signal transducer and activator of transcription 3 (STAT3) pathways [163]. Vinpocetine complements these actions by directly inhibiting IKK, preventing canonical NF-κB activation and promoting neural progenitor survival and differentiation [165]. The natural β-carboline alkaloid harmine suppresses neuroinflammation by downregulating the TLR4/NF-κB pathway and NLRP3-dependent inflammatory signaling [157].

#### 4.3.3. Antioxidant, Mitochondrial, and Pro-Survival Processes

Isoquinoline alkaloids like berberine activate pro-survival signaling cascades, including PI3K/Akt, AMPK, CREB, MAPK, and Nrf2, protecting mitochondria and lowering inflammation [166,167,168]. Berberine also elevates the activities of major antioxidant enzymes, including superoxide dismutase, glutathione peroxide, and catalase, thereby strengthening cellular defense against oxidative stress [169]. Sinomenine, an alkaloid extracted from the Chinese herb qingfenteng, was tested in the APP/PS1 transgenic AD mouse model and Aβ-42 oligopeptide-stimulated differentiated SH-SY5Y neuronal AD cell model. Oxidative stress, Aβ aggregation, and mitochondrial dysfunction were inhibited via the α7 nicotinic acetylcholine receptor (α7nAChR)/Nrf2/Keap1 pathway, indicating the α7 nicotinic acetylcholine receptor as a new target for AD prevention [170]. Rich alkaloid extracts from *Glaucium grandiflorum* (GGAE), including allocryptopine, tetrahydropalmatine, and tetrahydroberberine N-oxide, demonstrated their neuroprotective capacity by maintaining intracellular calcium homeostasis and dephosphorylating stress-related proteins such as ERK1/2, JNK, and p38 [153].

#### 4.3.4. Proteostasis and Autophagy Modulation

Alkaloids regulate proteostasis by inhibiting Aβ aggregation, modulating tau-related kinases, and promoting autophagic clearance. Novel harmine derivatives were reported to exhibit dual inhibitory activity against AChE and Aβ fibril formation at sub-micromolar potencies while maintaining low toxicity, highlighting their multi-target therapeutic potential [171]. Berberine, a natural alkaloid, has shown an impressive record in the inhibition of Aβ formation in previous studies, making it a potential candidate for the treatment of dementia-related diseases. Berberine counteracts ribosylation-driven Aβ pathology by suppressing the mTOR/p70 Ribosomal S6 Kinase (p70S6K) pathway, leading to improved spatial learning and memory in a widely used transgenic AD mouse model that carries human mutations in the APP and presenilin-1 (PS1) [172]. It also activates autophagy through the class III PI3K/beclin-1 signaling to facilitate the autophagic removal of tau protein in the triple-transgenic mouse model of AD (3 × Tg AD) [173]. Immunofluorescence data reported by Sun et al. revealed that berberine treatment in the five familial AD mutation (5 × FAD) mice and Aβ-induced SH-SY5Y cell models resulted in attenuation of Aβ plaque aggregation, suppression of autophagy, and induction of ferroptosis via the JNK-p38MAPK pathway [174]. GGAE alkaloid extract stimulates the phosphorylation of Akt and GSK-3β and concurrently ameliorates the phosphorylation of Tau, suggesting the association of the Akt/GSK-3β/Tau pathway in the treatment of neuroprotective diseases [153].

### 4.4. Biological Pathways of Terpenoids in Dementia-Related Diseases

Terpenes and terpenoids comprise the largest single class of plant secondary metabolites, with over 80,000 structurally characterized compounds built from repeating C5 isoprene units [175], and this structural diversity is matched by extensive pharmacological diversity. Diverse terpene and terpenoid classes, including small volatile monoterpenes, glycosylated triterpene saponins, and carotenoid polyterpenoids, affect multiple molecular targets implicated in AD and related dementias. Preclinical evidence supports terpene and terpenoid activity across the major pathological hallmarks of ADRD, and a subset of compounds, most notably the ginkgo diterpene trilactones and saffron carotenoids, have demonstrated cognitive benefits in replicated randomized controlled trials [12,47,176,177,178,179].

#### 4.4.1. Inhibition of Aβ Aggregation and Promotion of Clearance

BACE-1 is a key factor in amyloidogenic APP processing, and its inhibition reduces Aβ generation upstream of aggregation. Ginkgolides from *Ginkgo biloba* reduce BACE-1 activity and limit Aβ production [180,181]. Ginsenoside Rg1 from *Panax ginseng* reduces Aβ generation by suppressing BACE-1 gene expression rather than directly inhibiting the enzyme, a mechanism mediated by PPARγ nuclear translocation [182], while cryptotanshinone, an abietane diterpenoid from *Salvia miltiorrhiza*, reduces Aβ production by upregulating α-secretase activity through the PI3K pathway, shifting APP processing toward the non-amyloidogenic route [183]. Notably, several of the GSK-3β inhibitory effects described below further reduce BACE-1 transcription as a secondary effect, linking the tau and amyloid arms of the pathological cascade through a shared regulatory pathway [183].

At the level of fibril assembly, ginkgolide B, the diterpene trilactone component of EGb 761, inhibits Aβ fibril formation through direct molecular interactions [180]. Crocin, the water-soluble carotenoid from *Crocus sativus*, similarly inhibits Aβ fibrillogenesis in vitro [184]. Bacosides, the triterpenoid saponins of *Bacopa monnieri*, act at multiple points: they inhibit both BACE-1 and Aβ fibrillation and additionally reduce cortical Aβ levels in transgenic AD mouse models [47,185]. Withanolides from *Withania somnifera*, when administered orally in APP/PS1 and APPSwInd transgenic mice, significantly increased hepatic low-density lipoprotein receptor-related protein 1 (LRP1) expression and plasma-soluble LPR1. This LRP1 reservoir may serve as a peripheral sink to accelerate Aβ clearance from the brain [186].

#### 4.4.2. Suppression of Tau Hyperphosphorylation

Tau hyperphosphorylation in AD is driven primarily by the dysregulated kinases GSK-3β and CDK5, and PI3K/Akt-mediated inactivation of GSK-3β (via phosphorylation at Ser9) is a common mechanism across multiple terpenoid classes. Ginsenoside Rg1 decreases tau hyperphosphorylation and reduces GSK-3β expression in AD models via the Wnt/GSK-3β/β-catenin signaling pathway [187]. Bacosides reduce tau hyperphosphorylation and preserve microtubule integrity in Aβ42-injected rat models via the same GSK-3β interaction [47]. Cryptotanshinone reduces tau hyperphosphorylation through PI3K/Akt-mediated GSK-3β inactivation, along with improvements in spatial memory in scopolamine-induced amnesia models [183]. Withanolides from *Withania somnifera* (ashwagandha) attenuate tau pathology [47], and asiaticosides from *Centella asiatica* reduce tau hyperphosphorylation via GSK-3β inhibition and protein phosphatase 2A (PP2A) activation in AD rat models [188].

Saffron and its carotenoid constituents have demonstrated improvements in cognitive function and biomarkers in clinical trials of mild-to-moderate AD, although there is limited direct evidence that tau effects are the primary mechanism [177,178,189]. Ursolic acid, a pentacyclic triterpene widely distributed in medicinal plants including rosemary, demonstrates tau-directed activity through mechanisms that may involve CDK5 [183], providing an additional tau-directed mechanism that operates independently of GSK-3β.

#### 4.4.3. Attenuation of Neuroinflammation

Chronic neuroinflammation, driven largely by activated microglia and astrocytes, contributes to synaptic dysfunction, neuronal loss, and progression of AD pathology. Terpenoids have been reported to influence several inflammatory signaling pathways relevant to AD.

##### NF-κB Pathway Inhibition

Sustained NF-κB-driven microglial activation, with its linked production of TNF-α, IL-1β, and IL-6, amplifies Aβ deposition and accelerates neuronal loss in AD. Cryptotanshinone and tanshinone IIA from *Salvia miltiorrhiza* are among the most potent natural inhibitors of NF-κB-driven neuroinflammatory cytokine production described in the literature [183]. Ginsenoside Rg1 suppresses NF-κB signaling in neuroinflammatory models [190]. Bacosides suppress NF-κB-mediated neuroinflammation [47] and reduce pro-inflammatory cytokines including IL-6 and TNF-α in microglial models [47,191]. Ursolic acid inhibits NF-κB by blocking IκBα degradation and preventing p65 nuclear translocation [192], while withanolides suppress NF-κB signaling through targeting IKKβ [47].

Among the volatile terpenoids, 1,8-cineole (the dominant monoterpene of rosemary essential oil) and linalool (the principal constituent of lavender oil) reduce NF-κB-dependent gene expression via upstream kinase inhibition in cell models [52]. Linalool additionally potentiates gamma-aminobutyric acid A (GABA-A) receptors in electrophysiological assays [193] and inhibits glutamate binding at N-methyl-D-aspartate (NMDA) receptors in cortical membrane preparations [194]. Of mechanistic interest within this class are ginkgolide B and bilobalide from EGb 761, which suppress NLRP3 inflammasome assembly and downstream caspase-1 activation, a specific mechanism directly relevant to IL-1β processing in AD brain tissue that is not widely activated by other natural compounds [180,195].

##### CB2 Receptor-Mediated Microglial Modulation

β-Caryophyllene (BCP), a bicyclic sesquiterpene found at high concentrations in black pepper, cloves, and copaiba oil, is unique among terpenes and terpenoids studied for neuroinflammation because it is a selective agonist of the cannabinoid 2 (CB2) receptor [196], making it the only Generally Recognized as Safe (GRAS)-classified terpene by the U.S. Food and Drug Administration (FDA) with confirmed phytocannabinoid activity. Because CB2 is expressed primarily in microglia, its activation provides a receptor-mediated route to attenuating NF-κB-driven neuroinflammation, reducing pro-inflammatory cytokine release, and promoting microglial M2 polarization that is mechanistically distinct from the direct kinase inhibition described above [197]. In APP/PS1 transgenic mice, BCP administration reduced Aβ plaque burden, improved spatial memory in the Morris water maze, and reduced cortical inflammatory marker expression including COX-2, TNF-α, and IL-1β. These protective effects were reversed by the CB2 antagonist AM630 and the PPARγ antagonist GW9662, confirming the receptor-mediated and PPARγ pathway mechanism [198].

#### 4.4.4. Antioxidant Defense and Mitochondrial Protection

Oxidative stress and mitochondrial dysfunction are prominent features of AD and contribute to neuronal injury, impaired bioenergetics, and increased vulnerability to excitotoxic and inflammatory damage. Multiple terpenoid classes have been reported to influence antioxidant and mitochondrial pathways.

##### Nrf2/ARE Pathway Activation

Nrf2 is the master transcriptional regulator of cellular antioxidant defense, driving expression of HO-1, NQO1, glutathione peroxidase (GPx), SOD, and glutathione biosynthetic enzymes through the antioxidant response element (ARE). Its activity declines with age and is further compromised in the AD brain, creating an opportunity for pharmacological rescue. Withanolides from ashwagandha activate Nrf2 nuclear translocation, upregulating HO-1 and downstream antioxidant enzymes in microglial and cortical models [47]. Withaferin A, the most pharmacologically characterized withanolide, has similarly been shown to activate Nrf2/HO-1 signaling in neurodegeneration models [199]. Ginsenosides from *Panax ginseng* also contribute to antioxidant defense through pathways including Nrf2 activation and reduction in oxidative stress markers in AD models [47]. Asiaticosides from *Centella asiatica* induce antioxidant enzyme expression through Nrf2 activation in oxidative stress models [47]. The antioxidant effects of crocin are notable for having been supported by patient studies. In a randomized double-blind trial of saffron adjunctive to donepezil in mild-to-moderate AD, crocin-containing saffron elevated GPx, mitochondrial superoxide dismutase (SOD), and glutathione reductase activity and reduced total oxidative stress in clinical samples, providing human validation of the antioxidant response element (ARE)-dependent transcriptional program observed preclinically [200]. A common upstream mechanism across many of these terpenoid classes often involves PI3K/Akt or AMPK pathway activation, leading to Keap1-Nrf2 interaction disruption and Nrf2 nuclear translocation.

##### Mitochondrial Protection

Bilobalide, the sesquiterpene trilactone component of ginkgo extract EGb 761, is particularly well-characterized with respect to mitochondrial protection. It stabilizes mitochondrial membranes, upregulates NADH dehydrogenase gene expression in mitochondria, and modulates calcium channels to prevent excitotoxic calcium overload [180,201,202]. In cultured chick embryonic neurons and rat hippocampal mixed cultures, bilobalide rescues neurons from apoptosis induced by serum deprivation and by staurosporine and represents one of the most potent anti-apoptotic constituents in the EGb 761 extract [201]. In focal cerebral ischemia in gerbil models, oral bilobalide at 3–6 mg/kg/day for 7 days before transient global ischemia progressively protected CA1 neurons from death and preserved mitochondrial COX III mRNA levels, with effects comparable to the full EGb 761 extract [202].

Other terpenoid classes may also support mitochondrial integrity. Bacosides reduce mitochondrial oxidative damage and restore mitochondrial function in AD models [47]. Crocin has also shown neuroprotective effects in Aβ-exposed models, including reduction in neuronal injury in association with PI3K/Akt signaling [203].

#### 4.4.5. Modulation of Key Signal Transduction Pathways

##### PI3K/Akt/mTOR Pathway

The PI3K/Akt pathway is a central pro-survival cascade that promotes neuronal survival, inhibits apoptosis, regulates synaptic plasticity, and attenuates tau hyperphosphorylation through GSK-3β inactivation. Crocin activates PI3K/Akt signaling in Aβ25-35-injected mice, reducing neuroinflammatory cytokine production and hippocampal neuron injury [203]. Cornel iridoid glycoside (CIG) from *Cornus officinalis* activates the PI3K/Akt/mTOR pathway as its dominant proposed mechanism of action, improving memory performance in rodent models of both vascular dementia and AD, with corresponding reductions in hippocampal Aβ levels, tau phosphorylation, and neuroinflammatory markers [204]. Ginsenoside Rg1 engages PI3K/Akt as part of a broader neuroprotective program that extends through BDNF/TrkB signaling to support hippocampal neuroplasticity and long-term potentiation in APP/PS1 transgenic mice [190,205].

##### MAPK/ERK Signaling

The MAPK cascades, including p38, ERK1/2, and JNK arms, are relevant to both pro-inflammatory signaling and to neuroprotective and synaptic plasticity programs, depending on context and cell type. Saffron and its constituents crocin and safranal have demonstrated beneficial effects on cognitive outcomes in clinical trials of mild-to-moderate AD, with preclinical evidence supporting activity across multiple neuroprotective mechanisms [177,178,189]. Ginsenoside Rg1 engages ERK signaling through inhibition of ERK-mediated PPARγ phosphorylation, which in turn upregulates insulin-degrading enzyme (IDE) expression to promote Aβ degradation in a primary hippocampal neuron AD model [206].

##### GSK-3β Inhibition

GSK-3β occupies a key node in AD-related signaling because it contributes to tau phosphorylation and can influence amyloidogenic processing. Several terpenoid classes, including ginsenosides, bacosides, cryptotanshinone, and *Centella asiatica*-derived compounds, have been reported to reduce GSK-3β activity either directly or through upstream signaling pathways such as PI3K/Akt [47,183,187,188]. Thus, GSK-3β inhibition may provide a mechanistic link between tau-directed and amyloid-related effects, although the extent of this convergence differs among compounds and experimental models.

#### 4.4.6. BDNF Signaling and Neurotrophic Support

Loss of neurotrophic support is a major contributor to synaptic dysfunction and impaired plasticity in AD. BDNF and its receptor TrkB are particularly important for hippocampal long-term potentiation, neuronal survival, and memory formation. Ginsenoside Rg1 upregulates hippocampal BDNF and phospho-CREB in SAMP8 mice alongside dose-dependent reductions in soluble Aβ1-40 [207], and increases BDNF/p-TrkB protein expression with corresponding restoration of long-term potentiation in APP/PS1 transgenic mice [205]. Bacosides activate CREB-mediated neuroplasticity and support kinase-driven neuronal repair [47]. The withanolides function through a qualitatively distinct mechanism: withanolide A from ashwagandha promotes neurite outgrowth and synaptogenesis in cortical neurons through direct protein-level interactions, representing a potentially reconstructive effect rather than simply a neuroprotective one [208].

Asiaticosides from *Centella asiatica* consistently promote axonal elongation in culture models, and *Centella asiatica* extract has demonstrated improvements in neuronal morphology and Aβ plaque load in transgenic AD mouse models [47]. CIG, the combined morroniside and loganin fraction from *Cornus officinalis*, promotes nerve growth factor (NGF) and BDNF expression to support cholinergic neuron survival in fimbria-fornix lesion models of AD-like cholinergic deficit [204], a neurotrophic mechanism that is not activated by most other terpenoid classes and is relevant to the cholinergic neuronal loss central to AD. At the synaptic level, ginkgolides protect against Aβ1-42-induced synaptotoxicity and preserve synaptic protein expression in vitro [209], while a ginkgolide preparation also reduced Aβ deposition and neuroinflammation in transgenic mouse models [195].

#### 4.4.7. Promotion of Adult Neurogenesis

Adult hippocampal neurogenesis is substantially reduced in AD patients and transgenic AD models, and this reduction correlates with memory impairment. Compared with amyloid, tau, or inflammatory mechanisms, evidence for terpenoid effects on neurogenesis is less extensive, but several classes converge on signaling pathways relevant to neuronal differentiation, survival, and synaptic maturation.

Ginsenoside Rg1 promotes hippocampal neuroplasticity through BDNF/TrkB signaling, restoring long-term potentiation in APP/PS1 transgenic mice [205], and upregulating phospho-CREB and BDNF in SAMP8 mice [207]. Bacosides support neurogenesis through BDNF/CREB-mediated pathways [47]. *Centella asiatica* extract has demonstrated improvements in neuronal morphology in transgenic AD mouse models [47], with neurotrophic effects also documented in healthy elderly human volunteers [210]. The neurogenesis evidence for terpenoids is generally less extensively characterized at the mechanistic level than for flavonoids, but the convergence of ginsenosides, bacosides, and Centella-derived asiaticosides on BDNF/CREB and PI3K/Akt pathways that regulate neuroprogenitor survival and differentiation provides a plausible biological basis for these effects.

#### 4.4.8. Inhibition of AChE and Augmentation of Cholinergic Transmission

The progressive degeneration of basal forebrain cholinergic neurons is a major driver of the episodic memory and attentional deficits in AD, and inhibition of AChE and BChE to preserve synaptic acetylcholine is the mechanism of all currently approved AD therapies. AChE and BChE inhibitory activity is notably widespread across terpenoid structural classes [211]. Among the monoterpenes, 1,8-cineole, α-pinene, linalool, safranal, citral, and citronellal have been reported to exhibit AChE and BChE inhibitory activity in vitro, though potency varies considerably across compounds and assay conditions [52,212,213]. The significance of 1,8-cineole’s cholinesterase inhibitory activity has been directly validated under human conditions. Moss and Oliver [214] measured plasma 1,8-cineole concentrations in healthy volunteers following ambient rosemary essential oil exposure and found a significant positive correlation with both speed and accuracy of cognitive performance. Although suggestive, this does not directly establish cholinesterase activity as the primary mechanism. Safranal also shows AChE inhibitory activity in vitro [215], providing a cholinergic complement to crocin’s carotenoid-mediated effects from compounds derived from the same botanical source.

Among the triterpene classes, ginsenosides support cholinergic neurotransmission and protect cholinergic neurons from Aβ-induced degeneration [47]. Ginkgo terpenoids modulate cholinergic neurotransmission indirectly through neurotransmitter release mechanisms rather than direct enzyme inhibition [180], and ursolic acid demonstrates AChE inhibitory activity in vitro [183]. It is noteworthy that the lemon balm monoterpene aldehydes citral and citronellal, which are the principal constituents of Melissa officinalis essential oil extracts, were shown in radioligand binding assays to displace [^3^H]-nicotine from nicotinic ACh receptors and [^3^H]-scopolamine from muscarinic ACh receptors in human cerebral cortical membranes, a cholinomimetic receptor pharmacology that operates independently of AChE inhibition [216]. Clinical trials of *Melissa officinalis* essential oil in dementia have produced mixed results, with one placebo-controlled randomized controlled trial (RCT) reporting reduced agitation [217] and another finding no significant benefit over placebo or donepezil [218].

### 4.5. Biological Pathways of Phenolic Components in Dementia-Related Diseases

Phenolic acids, including caffeic acid, ferulic acid, gallic acid, rosmarinic acid, and protocatechuic acid, exert multi-target neuroprotective effects relevant to AD and related dementias [219,220]. Their mechanisms span antioxidant defense, anti-inflammatory signaling, mitochondrial support, amyloid and tau modulation, and synaptic protection [51,221].

#### 4.5.1. Antioxidant Defense and Redox Homeostasis

Phenolic acids exhibit potent antioxidant activity due to their hydroxyl groups, making them powerful radical scavengers, which neutralize reactive oxygen species (ROS), including superoxide anions, hydroxyl radicals, and peroxynitrite [221]. A synthetic caffeic acid phenethyl ester 4-*O*-glucoside, FA-97, mitigates oxidative stress-induced neuronal apoptosis and alleviates scopolamine-driven cognitive deficits by activating the Nrf2/HO-1 signaling pathway [222]. Treatment with sesamol, a natural phenolic compound, helps maintain redox homeostasis, safeguarding mitochondrial function, enhancing antioxidant enzyme expression by activating the Nrf2 pathway and promoting its nuclear translocation in H_2_O_2_-exposed SH-SY5Y cells [223]. Administration of monomeric polyphenol berry extract not only reinstates the reduced levels of tyrosine hydroxylase and dopamine transporter but also diminishes the buildup of α-synuclein within the midbrain via the NF-κB/Nrf2/Bax pathway [224].

#### 4.5.2. Modulation of Neuroinflammation

Phenolic acids inhibit microglial overactivation by suppressing key inflammatory pathways such as NF-κB/MAPK (urolithin) [225], SIRT1/NF-κB (resveratrol) [226], PPARγ/NF-κB (phloretin) [227], ERK/JNK/p38 (gallic acid and resveratrol) [228], GSK3β/PTEN/PI3K/Akt (trahydrocurcumin) [229], SIRT3/SOD2 (trilobatin) [230] and COX-2 [231]. Rosmarinic acid, in particular, downregulates pro-inflammatory cytokines such as TNF-α and IL-1β, thereby preventing chronic neuroinflammation associated with cognitive decline [232]. Gallic acid markedly reduces nitric oxide, iNOS, and IL-1β expression in the substantia nigra of lipopolysaccharide-infused rats, further alleviating neuroinflammation [233]. In D-galactose–treated mice, sesamol not only enhanced the mRNA and protein expression of the antioxidant enzymes HO-1 and NAD(P)H:quinone oxidoreductase 1 (NQO1), but also lowered the serum concentrations of the pro-inflammatory cytokines TNF-α and IL-1β [223].

#### 4.5.3. Enhancement of Synaptic Plasticity, Neurotransmission, and Neurogenesis

Many phenolic acids block Aβ aggregation by directly binding to Aβ monomers or destabilizing β-sheet-rich fibrils. Caffeic and rosmarinic acids inhibit BACE1, the β-secretase enzyme responsible for initiating Aβ generation, resulting in decreased Aβ plaque formation. Additionally, ferulic acid disrupts Aβ oligomerization and protects synaptic structure from Aβ-induced toxicity [234]. Phenolic compounds modulate tau pathology through inhibition of GSK-3β and CDK5, the two principal kinases responsible for tau hyperphosphorylation [219]. The use of different combinations of ferulic acid, curcumin, and phosphatidylserine results in remarkable decreases in Aβ, IL-1 β, and phosphorylated tau in addition to the elevation of brain-derived neurotrophic factor (BDNF) level [235]. Propofol enhances synaptic signaling by upregulating synaptic proteins such as synaptophysin and postsynaptic density protein 95 (PSD-95), thereby alleviating the cognitive impairment observed in the offspring of pregnant C57BL/6 mice exposed to isoflurane-induced anesthesia [236].

Many phenolic acids, such as caffeic acid [58], ferulic acid [237], rosmarinic acid [238], and gallic acid [239], have exhibited significant inhibition of acetylcholinesterase activity. Phenolic-rich extract of *Pinus densiflora* not only enhances long-term potentiation (LTP) induction but also counteracts the LTP suppression caused by scopolamine (SCOP), a muscarinic receptor antagonist, and reduces AChE activity [240]. Vanillic acid has been shown to mitigate SCOP-induced deficits in learning and memory by safeguarding hippocampal cholinergic function, reducing oxidative damage, and supporting synaptic plasticity [241]. After screening thousands of Chinese traditional medicines, forsythoside A has emerged as the most potent inhibitor of Aβ deposition by interfering with the pre-formed fibrils, thereby extending the lifespan of the *Caenorhabditis elegans* AD transgenic models [242].

Phenolic compounds promote neurogenesis not only through their antioxidant properties, but also by re-establishing a supportive neurogenic microenvironment. In an amyloid-β_1–42_-induced Alzheimer’s mouse model, rosmarinic acid treatment significantly restored hippocampal neurogenesis markers such as Ki-67 and doublecortin (DCX) and improved social memory, indicating enhanced neuronal proliferation and differentiation [243]. In addition, caffeic acid alkyl esters have been shown to stimulate extracellular ERK1/2 and Akt serine threonine phosphorylation in the rat PC12 neuronal cells, thereby promoting neurotrophic effects such as cell survival and differentiation [244]. Jiang et al. demonstrated that gallic acid stimulates the proliferation and differentiation of NSCs through activation of the MAPK/ERK pathway [245]. Moreover, Yu Ding et al. found that gallic acid enhances hippocampal neurogenesis and alleviates cognitive deficits in APP/PS1 mice via the GSK3β–Nrf2 signaling axis [246].

#### 4.5.4. Mitochondrial Protection and Homeostasis

Phenolic acids stabilize mitochondrial membrane potential, preserve ATP synthesis, and inhibit cytochrome-c release [234]. By regulating mitochondrial ROS balance partly through activation of the AMPKs/PGC-1α/Sirt3 pathway, honokiol counteracts NaF-induced oxidative stress and mitochondrial dysfunction. This leads to the prevention of neuronal and synaptic damage as well as the resulting cognitive impairments [247]. Polyphenol, when combined with physical activities, can promote mitochondrial biogenesis through the activation of Peroxisome proliferator-activated receptor-gamma coactivator 1-α (PCG-1α) in age-related central nervous system (CNS) disorder [248]. Anti-apoptotic actions include upregulation of B-cell lymphoma 2 (Bcl-2), p-PI3K, p-Akt, p-GSK-3β, Microtubule-associated protein 2 (MAP2), and synaptophysin (SYN) concurrently with suppression of Bax and caspase-3 were observed in the diabetic mice treated with salidroside [249].

### 4.6. Biological Pathways of Phytosterols in Dementia-Related Diseases

Phytosterols are plant-derived sterol alcohols that share the tetracyclic core of cholesterol but carry additional alkyl or unsaturated substituents at C-24, giving rise to the major dietary members: β-sitosterol, stigmasterol, campesterol, and the algal sterols fucosterol and 24(S)-saringosterol [250,251]. The relatively minor structural differences observed in phytosterols result in functionally distinct interactions with membrane lipid microdomains, sterol-sensing receptors, and nuclear transcription factors that are directly relevant to neuronal cholesterol homeostasis. Phytosterols cannot be synthesized by mammals and are exclusively diet-derived, with the richest concentrations found in unrefined vegetable oils, nuts, seeds, and legumes [252]. Unlike cholesterol, which is synthesized within the brain and largely excluded from CNS entry by the blood–brain barrier, dietary and circulating phytosterols have been shown in mouse studies to cross the blood–brain barrier and accumulate in brain parenchyma [253,254].

Evidence that phytosterol levels in the brain affect cognitive health comes primarily from epidemiological studies. Mendelian randomization analyses using genome-wide association data from the International Genomics of Alzheimer’s Project and the UK Biobank found that higher circulating stigmasterol levels were associated with a reduced risk of AD, and a similar inverse association was identified for sitosterol, while campesterol and brassicasterol showed no significant effects; however, these associations were attenuated after adjustment for blood lipids [255]. Other population-based studies in elderly subjects found that baseline sitosterol levels were associated with cognitive impairment, and that longitudinal increases in stigmasterol, campestanol, and sitostanol over five years were associated with cognitive decline. Thus, phytosterols appear to have complex and sterol-specific relationships with circulating phytosterol levels and cognition, with some sex-specific effects observed [256,257]. These human studies provide an indication that some phytosterols may have promise for enhancing cognitive health; however, compared to other classes of phytochemicals, there is significantly less mechanistic data available to support this potential use.

#### 4.6.1. Modulation of Amyloidogenic APP Processing

The proteolytic cleavage of APP by BACE1 and γ-secretase is strongly favored within cholesterol-enriched lipid raft microdomains, where APP and presenilin-1 co-localize to drive Aβ production. Because phytosterols differ structurally from cholesterol at C-24, their incorporation into neuronal membranes alters raft composition and shifts APP processing toward the non-amyloidogenic α-secretase route [252].

Among the common dietary phytosterols, stigmasterol has the most extensively characterized anti-amyloidogenic activity. Studies in APP-overexpressing neuroblastoma cells and in mice fed stigmasterol-enriched diets showed that stigmasterol reduces Aβ generation by directly decreasing BACE1 enzymatic activity, suppressing transcription of all γ-secretase complex subunits, reducing cholesterol and presenilin-1 from detergent-resistant lipid raft fractions, and reducing BACE1 internalization to the endosomal compartments where amyloidogenic cleavage of APP takes place. In in vivo studies, both BACE1 and γ-secretase activities were reduced in brain tissue, and Aβ40 and Aβ42 levels fell correspondingly, without any change in total brain cholesterol. This suggests that the effect arose from membrane compositional changes rather than bulk sterol displacement [252]. β-Sitosterol similarly promotes non-amyloidogenic APP processing by redistributing APP from lipid rafts to non-raft membrane regions, and treatment of APP/PS1 double transgenic mice improved spatial learning and recognition memory, reduced plaque load, and reversed hippocampal synaptic deficits normally observed in this model [252,258]. Complementary sterol profiling of aged APP transgenic mouse brain demonstrated that sterol metabolism is altered relative to non-transgenic controls, consistent with the proposition that amyloidogenic APP processing is coupled to broader dysregulation of brain sterol homeostasis [259].

Fucosterol, the predominant sterol in brown algae, inhibits BACE1 through non-competitive enzyme kinetics in vitro, with molecular docking analysis indicating interaction with residues of the BACE1 active site [260]. Pharmacological studies of fucosterol also identified liver X receptor β (LXR-β), TrkB, and TLR2/4 among its predicted targets, suggesting multiple mechanisms of neuroprotection [261]. In silico docking studies of β-sitosterol and stigmasterol isolated from *Polygonum hydropiper* likewise showed favorable binding geometries against BACE1 and monoamine oxidase, though these computational findings await experimental confirmation [262].

#### 4.6.2. Attenuation of Neuroinflammation

##### AMPK/NF-κB and NLRP3 Pathway Inhibition by Stigmasterol

Chronic microglial activation driven by NF-κB and NLRP3 inflammasome signaling amplifies Aβ deposition and accelerates synaptic injury throughout AD progression. Stigmasterol suppresses these pathways through AMPK activation, an upstream energy-sensing kinase whose activity dampens NF-κB nuclear translocation and NLRP3 inflammasome assembly. In APPswe/PS1dE9 transgenic mice, stigmasterol treatment attenuated cognitive deficits and reduced cortical and hippocampal Aβ42 concentrations. In BV2 microglia stimulated with Aβ42 oligomers, stigmasterol suppressed pro-inflammatory cytokine release and lowered M1 microglial polarization through this AMPK-dependent mechanism, thus reducing caspase-1 activation and IL-1β processing [263]. The engagement of an upstream metabolic sensing pathway distinguishes stigmasterol’s anti-inflammatory mechanism from the direct kinase inhibitory mechanisms of many flavonoid and terpene compounds.

##### Anti-Inflammatory Effects of β-Sitosterol

β-Sitosterol modulates neuroinflammatory signaling through several complementary mechanisms. Dietary studies in a murine model showed that a plant sterol-poor diet was associated with elevated pro-inflammatory lipid mediators in the brain, with thromboxane B2 and prostaglandin D2 concentrations inversely correlated with campesterol and β-sitosterol levels across brain regions. This suggests that endogenous phytosterol levels in the brain may reduce neuroinflammatory signaling [264]. In a cardiovascular disease model, β-sitosterol suppressed MAPK pathway signaling [265] and NLRP3 inflammasome activation and upregulated antioxidant enzymes through Nrf2 [266]. Although these findings derive from non-CNS models, the involvement of MAPK and NLRP3, both implicated in AD neuroinflammation, provides a plausible mechanistic basis for analogous effects in the brain.

##### Gut–Brain Axis Modulation

An emerging potential mechanism through which phytosterols may reduce neuroinflammation involves modulation of the gut–brain axis. Gut microbiota dysbiosis, characterized by reductions in short-chain fatty acid (SCFA)-producing bacterial populations, has been linked to increased Aβ deposition, tau hyperphosphorylation, blood–brain barrier disruption, and heightened neuroinflammation in AD [265]. Phytosterols have been proposed to partially correct this dysbiosis by promoting SCFA-producing bacteria, resulting in increased secondary bile acids that signal through the Farnesoid X receptor (FXR) and Takeda Gprotein-coupled receptor 5 (TGR5) receptors to suppress neuroinflammatory tone. The preclinical AD model evidence reviewed by Ganamurali et al. [265] supports phytosterol-driven gut–brain axis effects, although direct mechanistic studies specifically identifying the gut microbiota contribution to phytosterol-mediated CNS outcomes remain limited.

#### 4.6.3. Antioxidant Defense and Membrane Protection

##### PI3K/GSK-3β Signaling and Membrane Antioxidant Effects

β-Sitosterol incorporated into neuronal plasma membranes protects against oxidative stress and lipid peroxidation through estrogen receptor-dependent signaling. Studies in hippocampal cell lines and primary hippocampal neurons demonstrated that membrane-bound β-sitosterol activates PI3K and recruits it to lipid rafts, leading to downstream phosphorylation and inactivation of GSK-3β. Estrogen receptor antagonism or PI3K inhibition abolished these protective effects, confirming receptor-dependent signal transduction [267]. The dual effects—the reduction in oxidative membrane damage in concert with GSK-3β inactivation—are particularly relevant to AD, where lipid peroxidation is elevated from early disease stages and GSK-3β drives tau hyperphosphorylation.

##### Nrf2 Pathway Activation

There is little evidence that phytosterols affect this important pathway in the development of ADRD. Stigmasterol has been shown to upregulate Nrf2 expression and attenuate oxidative stress in a rheumatoid arthritis chondrocyte model [268], while in the ApoE−/− cardiovascular model, β-sitosterol activated Nrf2 and upregulated antioxidant enzyme activity [266]. Further studies are required to determine if these results translate into a contribution to neuroprotection in cognitive decline.

#### 4.6.4. LXR Activation and Cholesterol Homeostasis

Liver X receptors (LXRα and LXRβ) are nuclear cholesterol sensors whose activation in the brain upregulates ATP-Binding Cassette Transporter A1 (ABCA1) and ATP-Binding Cassette Transporter G1 (ABCG1) cholesterol transporters, increases apolipoprotein E (ApoE) secretion from astrocytes and microglia, and promotes cholesterol efflux from neurons [269]. The lipidation state of ApoE governs whether it promotes Aβ clearance or aggregation, and ApoE itself is an LXR target gene. Thus, LXR-mediated cholesterol redistribution provides a potential pathway for reducing amyloid burden.

Several phytosterols, including β-sitosterol, stigmasterol, and fucosterol, have been identified as LXR ligands in cell-based reporter systems, though systematic screening suggests that the major dietary phytosterols at physiological plasma concentrations have limited capacity to activate LXRs [270]. The algal oxyphytosterol 24(S)-saringosterol from *Sargassum fusiforme* is, by contrast, a potent selective LXRβ agonist. Dietary supplementation with *Sargassum fusiforme* or its lipid extract improved short-term memory, substantially reduced hippocampal Aβ plaque load in APPswePS1ΔE9 mice, and activated LXR-responsive genes in brain tissue without inducing hypertriglyceridemia or hepatic steatosis [270]. A subsequent study using purified 24(S)-saringosterol showed that it prevented cognitive decline and reduced microglial Iba1 expression in the cortex of APPswePS1ΔE9 mice; however, this occurred without a significant effect on Aβ plaque load, suggesting that the full neuroprotective activity of the seaweed extract may require contributions from multiple constituents that work in concert with 24(S)-saringosterol [271]. The semi-synthetic derivative 22-ketositosterol has also shown LXR agonism and has been reported to slow AD progression in transgenic mouse models [272].

#### 4.6.5. Inhibition of Acetylcholinesterase and Augmentation of Cholinergic Transmission

Progressive loss of basal forebrain cholinergic neurons and the resulting acetylcholine deficit are core features of AD. Multiple phytosterols have demonstrated cholinesterase inhibitory activity in experimental systems, suggesting mechanistic overlap with existing AD treatments.

Stigmasterol has been evaluated in a scopolamine-induced amnesia model, in which oral administration reversed memory impairment in passive avoidance and Morris water maze tasks. Hippocampal ERK1/2 and CREB phosphorylation were elevated in stigmasterol-treated animals, and the cognitive effects were abolished by NMDA-receptor and estrogen receptor antagonism, indicating that memory enhancement proceeds through NMDA and estrogen receptor-linked signaling rather than simple cholinesterase inhibition alone [273].

β-Sitosterol isolated from *Polygonum hydropiper* inhibited both AChE and BChE in vitro. In transgenic mice, cholinesterase inhibitory activity was also observed in the frontal cortex and hippocampus and was correlated with behavioral improvement. Molecular docking further supported the direct binding of β-sitosterol to the active sites of both enzymes [274]. Fucosterol has also demonstrated moderate AChE inhibitory activity, consistent with network pharmacology predictions identifying AChE as a potential significant binding target [261].

#### 4.6.6. Modulation of Key Signal Transduction Pathways

##### PI3K/Akt/GSK-3β Pathway

β-Sitosterol activates PI3K/Akt through an estrogen receptor-dependent membrane-mediated mechanism, resulting in downstream GSK-3β inactivation [267]. Since GSK-3β phosphorylates tau at multiple disease-relevant epitopes and can upregulate BACE1 transcription, its inactivation provides a potential point of intersection between phytosterol membrane effects and both tau- and amyloid-related pathology. However, unlike several flavonoids and terpenoids, current phytosterol studies do not directly demonstrate reduced tau phosphorylation or inhibition of tau fibril formation. Network pharmacology analysis of fucosterol similarly identified PI3K/Akt signaling as a primary enriched pathway, alongside TNF and MAPK cascades [261]. More studies are needed to confirm and define the effects of phytosterols on this critical pathway.

##### ERK/CREB Signaling

The stigmasterol-dependent upregulation of ERK1/2 and CREB phosphorylation in the hippocampus of the scopolamine amnesia model demonstrates potential phytosterol involvement in memory-consolidating signal transduction downstream of NMDA receptor and estrogen receptor activation [273]. This ERK/CREB cascade overlaps with the PI3K/Akt and BDNF/TrkB signaling described for flavonoids and terpenes in the preceding sections and represents a recurring convergence point across diverse phytochemical classes that activate neuroprotective and synaptic plasticity gene expression. Network pharmacology predictions identified TrkB as a predicted target of fucosterol [261], suggesting that algal phytosterols may support neurotrophic signaling, although experimental validation in cell or animal models is required.

### 4.7. Summary of the Phytochemical Compounds in Section 4

**Figure 3:**
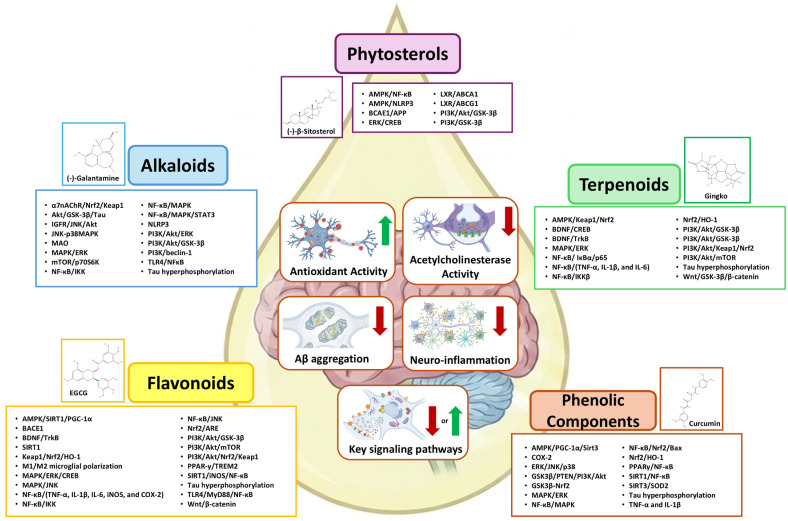
Summary of key signaling pathways involving five major phytochemical groups: alkaloids, flavonoids, terpenoids, phenolic components, and phytosterols. All five groups have demonstrated the ability to enhance antioxidant activity and to inhibit Aβ aggregation, neuroinflammation, and acetylcholinesterase activity. These phytochemicals also modulate key signaling pathways (see text for details). For each phytochemical group, the compound with the most advanced clinical evidence or the most compelling translational profile is shown. Chemical structures were retrieved from the PubChem database (1 May 2026 https://pubchem.ncbi.nlm.nih.gov/) (Partially created using FigureLab, https://mbaffour.github.io/FigureLab/figure_lab.html, accessed on 1 May 2026).

Table 5 and Figure 3 summarize the examples of phytochemical compounds described in Section 4. These are only examples among the broad range of phytochemical compounds studied. Other than being included in essential oils, many of them are included in tea (for example, EGCG), and there are many available as supplements as well (for example, quercetin).

## 5. Studies on the Effects of Essential Oils on Dementia

### 5.1. Observations of the Effects of Essential Oils on Nursing Home Patients by Aromatherapists

Reports by aromatherapists who have years of experience conducting, for example, hand massages on healthy clients and patients with dementia are important resources of information on the possible effects of the phytochemicals in essential oils. Table 6 summarizes examples of observations on reactions following hand massage using essential oils. These reports most likely include the effects of hand massage itself and the effects of interaction with the aromatherapist in addition to the effects of phytochemicals in the essential oils (unpublished data by KT and YM), and there could be synergistic influences of each factor as well.

### 5.2. Effects of Essential Oils on Cognitive Functions

Numerous studies have shown the effects of essential oils or their chemical constituents on improving cognitive function or neuropsychiatric symptoms. Woo et al. [275] showed that cognitive function increased by 226% in healthy seniors over 60 years in age who used a diffuser for two hours in the evening with seven different essential oils rotating daily [275]. Cognitive function was assessed using the Ray Auditory Verbal Learning test and the Weschler Adult Intelligence Scale (ver.3) (WAIS-III). The remarkable increase in cognitive function test scores suggests that essential oils can be a promising new agent for preventing the decline of cognitive functions in seniors. The study did not compare the effects of the seven essential oils used, nor did it analyze their chemical compositions, which can vary substantially even among oils with the same name. This information would be important to know whether all the essential oils were effective or, if not, which one caused the observed effects. The differences due to the types of essential oils, if any, are also important for determining which phytochemical compounds had significant effects in improving cognitive functions.

In another study, 28 patients with either AD, AD and cerebrovascular lesions, or vascular dementia were exposed to a mixture of lemon and rosemary essential oils in the morning (9:00 to 11 am) and a mixture of lavender and orange essential oils in the evening (7:30 pm to 9 pm) for 4 weeks using a diffuser system [276,277]. The effects were evaluated using the Functional Assessment Staging of Alzheimer’s disease (FAST), Hasegawa’s dementia scale (HDS-R), a Japanese version of the Gottfries, Braine, Steen Scale (GBSS-J) on cognitive function (GBSS-J-A), spontaneity (GBSS-J-B), feeling function (GBSS-J-C), other moral condition (GBSS-J-D), and movement function (GBSS-J-E). The study found that there was significant improvement in cognitive function, specifically in abstract function (GBSS-J-A-13), in the patients with mild to moderate AD [277].

Other than essential oils, there are some studies showing the effects of medicinal plants on improving cognitive function or memory [278,279]. In a review study by Kirubakaran [278], lavender (*Lavandura angustifolia*) and common sage (*Salvia officinalis*), which are well-known for their essential oils, were listed as herbal remedies for treatment of AD. In addition, various herbs and spices, which are not usually used as essential oils, were also listed to improve cognitive function: for example, ginkgo (*Ginkgo biloba*), waterhyssop (*Bocopa monnieri*), rhodiola (*Rhodiola rosea*), and fir moss (*Huperzia serrata*) [278]. Another study using extract drops of lemon balm (*Melissa officinalis*) showed improved cognitive function (measured by the Alzheimer’s disease assessment scale (ADAS-cog)) after four months without side effects [280].

Indian pennywort (or gotu kola, Asiatic pennywort, Ji Xue Cao, *Centella asiatica*) is well-known as one of the medicinal plants used in Ayurveda [281] (a traditional medical system of India) [282] and in traditional Chinese medicine [283]. In a review, Sun et al. [283] reported that *Centella asiatica* improved cognitive functions in studies using mice or rats as animal models. Wattanathorn et al. [210] conducted a clinical study in which 28 healthy seniors (average age 65) took a capsule that contained either 250, 500, or 750 mg (or a placebo) of extract of *Centella asiatica* daily for 8 weeks. They found that participants who took *Centella asiatica* showed faster reaction times and higher accuracy in cognitive function tests after the 8 weeks [210]. There is also currently an ongoing Phase I clinical study in the U.S. by Soumyanath et al. (accessed on 11 March 2026, https://clinicaltrials.gov/study/NCT05591027) testing the effects of taking *Centella asiatica* for 6 weeks, indicating growing interest in the effects of *Centella asiatica*. Pharmacokinetic and pharmacodynamic studies of the group also showed that the aqueous extract of *Centella asiatica* was safe [11].

Waterhyssop (*Bacopa monnieri*) is another herb used in Ayurveda. Essential oil derived from *B. monnieri* is called bacopa oil or brahmi oil. A clinical trial in Australia tested the effects of waterhyssop in 101 participants (age 40 to 70 with self-reported memory and attention problems; in total, 40 participants who took 300 mg of *B. monnieri* extract daily and 47 participants who took placebo completed a 12-week period). Although there were no significant differences in the cognitive test performances, the self-reported stress level and fatigue after the cognitive tasks were significantly lower [284].

Another plant well-known as one of the medicinal plants used in Ayurveda [281] and traditional Chinese medicine [283] is the false daisy (other names of the same plant: bhangra, bhringaraj, or Han Lian Cao, *Eclipta prostrata,* or *E. alba*). We are not aware of any clinical trials on its effects on dementia yet, although there are some studies using rats as animal models showing that it improved learning and memory [285].

### 5.3. Effects of Essential Oils on Neuropsychiatric Symptoms

There have been studies on utilizing essential oils for treating neuropsychiatric symptoms [217,280,286,287], in which lemon balm (*Melissa officinalis*) [217,280] and lavender (*Lavandula angustifolia*) [287] were used, showing positive effects on suppressing agitation. In these studies, Ballard et al. [217] used lemon balm essential oil by mixing it into a base lotion (sunflower was used as the placebo for the control group) and applying it on the face and arms of the patients twice a day for four weeks [217]. Agitation (evaluated using the Cohen-Mansfield Agitation Inventory (CMAI)) and Quality of Life Indices (measured by Dementia Care Mapping) both showed improvement in the group treated with lemon balm essential oil. In the study by Lin et al. [287], diffusers were used. Two drops of pure lavender (*Lavandula angustifolia*) certified by the Aromatherapy Organization Council (sunflower was used in the control group) were put on cosmetic cotton and placed in diffusers, and two diffusers were placed on each side of the pillows of the patient at night for at least 1 h [287]. Effects were evaluated after three weeks using the Chinese version of the Neuropsychiatric Inventory (CNPI) and CMAI, and significant improvements in both CNPI and CMAI were observed. The treatment’s effects were especially significant for agitation, irritability/lability, aberrant motor behaviors, and night-time behaviors [287]. There are some studies using lavender that did not show measurable effects [288], which could be due to differences in methods or the essential oil chemical profiles, and further tests will be required.

Other than bergamot and lavender, numerous candidate essential oils have been tested using animal models and in human studies, not necessarily related to dementia but more focused on depression and anxiety [289]. Sweet orange (*Citrus sinensis*), yuzu (*Citrus junos*), lemon (*Citrus limon*), chamomile (*Matricaria chamomilla*), rosemary (*Salvia Rosmarinus*), sage (*Salvia officinalis*), Spanish sage (*Salvia lavandulaefolia*), Japanese sweet flag (*Acorus gramineus*), perilla (*Perilla frutescens*), coriander (*Coriandrum sativum*), asarum (*Asarum caudatum*), rose (*Rosa disambiguation*), and ylang-ylang (*Cananga odorata*) are listed as having effects on increasing serotonin secretion and locomotor activity in animal model studies and antidepressant effects and anxiolytic effects in human studies [289]. These studies suggest that there are many candidate essential oils that may have effects on dementia but are still in need of testing.

As briefly mentioned in Section 3.2.3, drugs for neuropsychiatric symptoms such as Brexpiprazole can increase the risk of stroke, resulting in a higher chance of death [41,42,43]. The development of methods that have fewer risks is important, and the possibility of fewer life-threatening adverse events using essential oils suggests that they may offer a promising alternative to these drugs, which have an increased risk of death (see Section 6.2 for studies on improving the stability of and reducing the adverse events caused by some essential oils).

### 5.4. Effects of Essential Oils on Factors Associated with Dementia

There are many studies testing the effects of each phytochemical compound on Aβ, AChE, etc. Table 7 shows examples of the essential oils or the plants that showed effects on factors associated with dementia, either in clinical studies or animal model studies. This suggests there is a high possibility of identifying the phytochemical compounds, and plants rich in them, which can become future drugs for dementia. It is also possible to see from the table that the same plant/essential oils have multiple effects. This is not surprising considering that each disease mechanism (such as Aβ, tau, AChE, etc.) is not an independent factor, and considering that each plant has hundreds of chemical constituents and that multiple constituents often possess bioactive properties. The large number of plants in these examples indicates the difficulties in going through an extensive number of plants to identify the best candidate(s) for further study. To facilitate this process, we propose a network machine learning strategy (see Section 6.1), in which the mechanisms of action of phytochemical compounds can be compared to the drugs for dementia to identify the plants that contain many of them.

### 5.5. Phytochemical Compounds in the Essential Oils Can Reach the Brain Through the Nose-to-Brain Pathway and the Transdermal Pathway

#### 5.5.1. Nose-to-Brain Phytochemical Pathway and the Pathway in Sensing Odors

**Figure 4:**
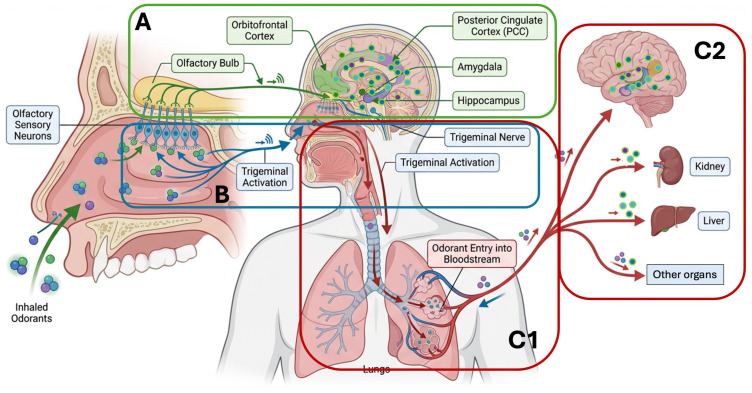
The pathways involved when a diffuser is used. (**A**) Olfactory pathway: Inhaled odorants enter the nasal cavity and activate olfactory sensory neurons. The signals reach the olfactory bulb, and from there, the orbitofrontal cortex, amygdala, hippocampus, and PCC. (**B**) Trigeminal pathway: Inhaled odorants also activate trigeminal nerves and reach the lung (**C1**). Once they enter the bloodstream, they reach various locations in the body, including the brain (**C2**). (Generated using FigureLabs, https://mbaffour.github.io/FigureLab/figure_lab.html, accessed on 1 May 2026).

Utilization of the nose to deliver a drug to the brain has been receiving increasing interest as it enables bypassing the blood–brain barrier (BBB), delivers the drug to the CNS quickly, and is noninvasive [314,315,316,317,318]. The route through the nose includes the olfactory pathway (Figure 4A), the trigeminal pathways (Figure 4B), and the respiratory pathway (Figure 4(C1,C2)) [319], among which the nose-to-brain pathway utilizes the olfactory pathway as a gate to the brain. There are recent studies trying to establish this method as a method to deliver drugs to patients with dementia as an alternative to IV injections or pills [320,321,322]. This indicates that the inhalation of an essential oil either from a diffuser or when it is used in a hand massage can also deliver the phytochemicals contained in it from the nose to the brain, i.e., nose-to-brain phytochemical delivery. Thus, the phytochemicals that have bioactive properties that are beneficial in treating dementia can reach the brain, bypassing the BBB, in a fast and noninvasive way.

A key question is where in the brain these phytochemicals reach via nose-to-brain drug delivery and how they do so. A confusing fact is that, as they have smells, we tend to misunderstand that the mechanisms of our olfactory sense, which we use in detecting, recognizing, and memorizing smells by the activation of olfactory receptors on olfactory sensory neurons (Figure 4A locations written in green boxes), are involved. The route of olfaction is based on the activation of olfactory receptors, which does not require the transport of the chemical compounds that activate the olfactory receptors. The activated signals reach the olfactory bulbs, and from there, the orbitofrontal cortex (olfactory cortex) for recognition, the amygdala to affect hormone secretion, and the hippocampus for memorization.

**Figure 5:**
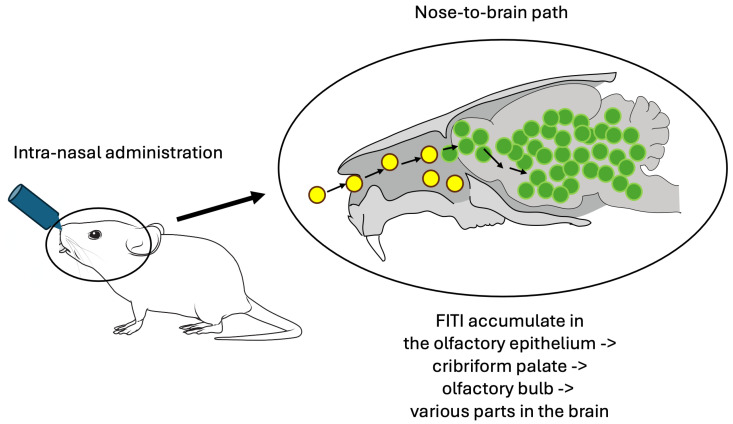
Illustration of the dispersal of FITC after intranasal administration of commensal bacteria loaded with FITC. FITC was released from the bacteria and dispersed in various parts of the brain (prepared based on the description in Shen et al. [317]). (Illustration of mouse by Ethan Tyler and Lex Kravitz (https://doi.org/10.5281/zenodo.3926069) and of the mouse skull by Ann Kennedy (https://doi.org/10.5281/zenodo.3925963); both from SciDraw.io (https://scidraw.io, accessed on 15 May 2026)).

How phytochemical compounds are delivered from the nose to the brain needs to be considered separately from how the smells are detected. This is critically important because we need the phytochemicals to be ‘delivered’ to the areas where there is neuroinflammation, aggregation of Aβ, and accumulation of other enzymes and proteins related to dementia, causing negative effects on neuronal health. The details on how phytochemicals become transported through the nose-to-brain pathway are not well known. However, in a recent study by Shen et al. [317], the authors used mice as an animal model system and administered commensal bacteria labeled with fluorescein isothiocyanate (FITC) to the nasal cavity. It was known that FITC would be released gradually from the bacteria, with the peak release within 48 h. This allowed them to trace the travel of FITC after the commensal bacteria were administered intranasally. They found that FITC traveled through the olfactory nerve bundles in the lamina propria under the olfactory epithelium, from there to the olfactory bulb, and eventually to broad regions in the brain [317] (Figure 5). In another study using mice as an animal model, Oguro et al. [323] exposed mice to linalool (4ppm as a mist) for 2 h and measured the levels of linalool in the brain and in the other organs of the mice using GC-MS. Linalool was found in the olfactory bulb, cerebral cortex, cerebellum, lung, and blood [323], showing that phytochemical compounds can reach various locations inside the brain within two hours by inhalation.

#### 5.5.2. Transdermal Pathway

**Figure 6:**
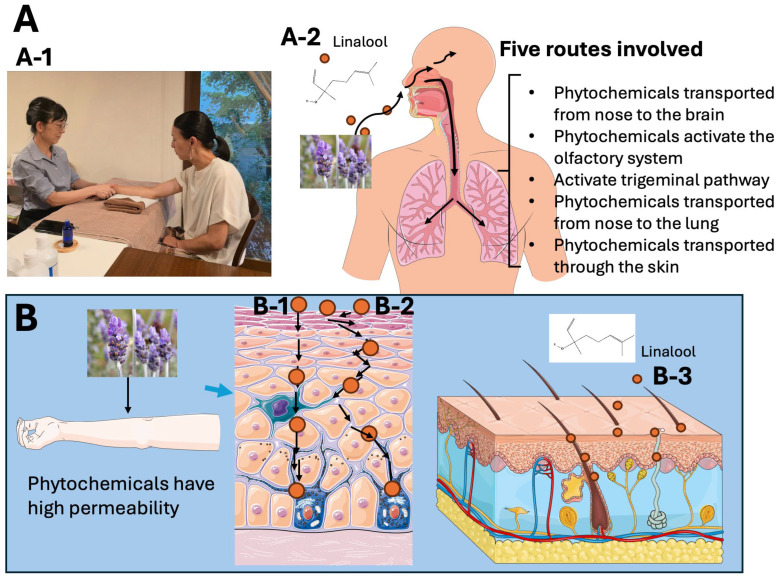
The routes of phytochemical compounds involved in hand massage. (**A**) Typical hand-massage treatment view (**A-1**). (**A-2**) Five routes are involved. Phytochemicals can be inhaled and the odorants enter the nasal cavity, similarly to the pathways shown in Figure 4, and (1) reach the brain in the nose-to-brain pathway, (2) activate the olfactory sense, (3) activate the trigeminal pathway, (4) reach the lung and enter the blood system and from there, the brain, and (5) permeate the skin and enter the blood system and from there, the brain. (**B**) Phytochemicals applied to skin by hand massage can pass through the skin by three routes: (**B-1**) intracellular, (**B-2**) intercellular, and (**B-3**) through hair follicles and sweat ducts. Thus, they can reach the brain through the nasal pathway and through the bloodstream. Once they enter the bloodstream, they can reach various locations in the body as well. (Images of the nasal cavity, brain, and skin were provided by Servier Medical Art (https://smart.servier.com), licensed under CC BY 4.0 (https://creativecommons.org/licenses/by/4.0/, accessed on 5 May 2026).).

When essential oils are applied by hand massage, multiple routes are involved (Figure 6A). As they are highly volatile, we can smell the odor of the essential oils when they are applied to the skin, which indicates that we inhale the phytochemical compounds contained in the oils. This allows the routes through the nose as already described above (see Section 5.5.1). Here we will focus on the transdermal pathway.

Essential oils are well-known to permeate skin easily, and they are even used as one of the methods of permeation enhancers in transdermal drug delivery [324,325,326,327]. There are three routes involved in the transdermal pathway (Figure 6B): intracellular (Figure 6(B-1)), intercellular (Figure 6(B-2)), and through the hair follicle and sweat gland ducts (Figure 6(B-3)). Importantly, when essential oils are used in hand massage, they are diluted using carrier oils [328,329] to avoid contact dermatitis [330,331] and evaporation, as well as to maintain/manage the concentration. Oils from jojoba (*Simmondsia chinensis*), almond (*Prunus amygdalus*), seeds of grape (*Vitis vinifera*), coconut (*Cocos nucifera*), apricot (*Prunus armeniaca*), and calendula (*Calendula officinalis*) are often used as carriers. The dilution rates recommended by the Japan Aromacoordinator Association (JAA) are 2 to 3%, which depends on the types of essential oil and also on the types of carriers [329]. Diluting essential oils may trigger questions about concentration and effects. In a study by Komori et al. [328], a mixture of three different essential oils diluted with jojoba oil was used. The dilution rates of the essential oils to jojoba oil were 1:150 to 1:300 (0.33% to 0.67%): lemon (*Citrus limon*) (0.67%), tuberose (*Polianthes tuberosa*) (0.33%), and labdanam (*Cistus ladanifer*) (0.33%). They asked the participants to go through the Uchida-Kraepelin Performance test for 30 min, and then the participants were provided with either a hand massage with jojoba oil without essential oil, a full hand massage with essential oil, a simpler hand massage with essential oil, or no treatment as a control. Though the dilution rates they used were much lower than those recommended by the JAA, they still found significantly reduced stress levels in the participants that received a hand massage with essential oils in both the full massage and simpler massage groups compared to the no-treatment group and hand massage with only-carrier-oil group, which came out in the middle, between the no-treatment and essential oil groups [328].

## 6. Conclusions and Future Perspective

As we have summarized so far, there is a high potential for utilizing essential oils or plant-derived supplements and medicines for patients with dementia. One of the biggest obstacles in the case of essential oils could be the ‘image’ of essential oils that many people still have, i.e., that they are non-scientific (often using the word ‘holistic’ in that meaning) and that they are only for relaxing and enjoying the scents. Unfortunately, we receive these comments often, and we hear surprise that there is actually solid scientific research on the basic chemistry of phytochemical compounds. One of the goals of our review is to enhance the understanding of phytochemical compounds and their effects.

The more we know about phytochemical compounds and their bioactive properties, the more we notice there are several questions that need to be answered: for example, (1) which one should be used for what condition, (2) can we or should we improve the delivery by using a chemical formulation, (3) should we use them as a single chemical compound or as a combination or as a whole extract, and (4) what is the best delivery method? It must be emphasized that the phytochemical approaches and aromatherapy strategies discussed in this review are intended to be evaluated as complementary interventions alongside, not as replacements for, established pharmacotherapy. Patients receiving FDA-approved treatments for dementia should not discontinue or modify their medications based on preclinical or early-phase clinical evidence for plant-derived compounds without consultation with their healthcare providers.

### 6.1. Finding the Most Promising Plant for the Treatment or Prevention of Dementia: Network Machine Learning

The extensive number of medicinal plants and their extracts, with an even more extensive number of phytochemical compounds, is overwhelming. Which one(s) should be used is a difficult question to answer quickly, and we see papers on the effects of essential oils without the rationale on how/why the oils were selected. It would be extraordinarily helpful if we could select them based on scientific evidence on the phytochemical constituents.

In 2019, Veselkov et al. [332] conducted an extensive machine learning analysis of 7692 bioactive phytochemical compounds that possess the same mechanisms of action as the existing, clinically approved anti-cancer therapies [332]. Then they conducted an analysis of foods that contain these phytochemical compounds (naming them ‘Hyperfoods’ from their cancer-beating nature) [332]. Using the same methodology, they also determined the ‘Hyperfoods’ for COVID-19 [333]. In 2023, using the same methodology but focusing on olive oil, they determined the phytochemical compounds included in olive oil that have a high possibility of suppressing the onset and progression of AD [334]. Table 8 shows the top 10 phytochemical compounds included in olive oil identified in their study to have beneficial effects on the suppression or treatment of AD [334]. These approaches have a high possibility of providing rationales for selection from the broad number of plants if the analyses are expanded to include a large number of phytochemical compounds, as the authors did for their studies on cancer and COVID-19 [332,333].

### 6.2. Enhancing Stability and Reducing Toxicity

There are multiple methods for extracting essential oils, and depending on the methods, there are large differences in their chemical constituents [335,336,337,338,339]. Traditional methods include hydro-distillation, steam distillation, solvent extraction, pressured liquid extraction, and cold pressing extraction [335]. There are many recently developed methods which use, for example, enzyme-assisted extraction, fractional distillation, microwave-assisted hydro-distillation, solvent-free microwave extraction, and so on [335]. The differences in the chemical constituents depending on the distillation methods also affect the bioactive properties [335,337,338]. Mohanty et al. [338] used three different Curcuma plant species and compared the chemical constituents and the bioactive properties of their essential oils when hydro-distillation and solvent-free microwave extraction were used as extraction methods [338]. They found that, although the major chemical constituents were qualitatively the same, solvent-free microwave extraction produced more essential oil volume, with significantly different quantities, and the essential oils made using this method showed better results in their cytotoxicity [338]. These studies suggest that new technologies for making essential oils are more efficient than traditional methods.

New technologies also allow totally different strategies, for example, to stabilize unstable chemical constituents and eliminate furocoumarins, which can cause skin reactions called phytophotodermatitis, after topical application and exposure to UV [340,341]. Furocoumarins, or furanocoumarins, are tricyclic aromatic compounds found especially in citrus (Rutaceae) such as citrus fruits (bergamot, lime, lemons, etc.) and Apiaceae family plants such as carrots, celery, parsley, etc. [341,342]. Although they can cause phytophotodermatitis, they also have ‘photo-protective effects’ on terpenes, suppressing their degradation [343], which indicates that simply excluding furocoumarins does not provide a complete solution, because, if they are chemically excluded, it is necessary to protect the remaining chemical constituents from degradation.

As an attempt to solve this problem, Scuteri et al. [344] developed bergamot without bergapten [344,345], the major furocoumarin included in bergamot (bergapten-free bergamot, BEO-BF), which maintained the pharmacological properties of bergamot. In addition, in order to enhance the stability of the remaining chemical constituents, they used solid lipid nanoparticles (SLN), chemically encapsulating BEO-BF (NanoBEO) [344]. This chemically formulated bergamot without bergapten showed significant anti-nociceptive and anti-allodynic effects in their study using mice as an animal model [344]. Scuteri et al. [43] conducted a clinical trial with patients with dementia, applying NanoBEO on their arms once daily for four weeks, and determined that NanoBEO significantly reduced the frequency and level of agitation in the patients [43]. As such, new chemical strategies are being incorporated into or replacing the traditional methods, leading to a decrease in adverse events from the chemical constituents and improving the outcomes.

Another chemical compound with known toxicity at high doses is α-thujone. α-Thujone is found in plants such as common sage (*Salvia officinalis*) and wormwood (*Artemisia absinthium*) (Spanish sage, *Salvia lavandulifolia*, and sweet wormwood, *Artemisia annua*, contain low or non-detectable amounts of thujone). Although the concentration of α-thujone in common sage and wormwood is safe for culinary use, it is suggested that higher doses included in essential oils may cause serious health problems. Studies have shown that α-thujone causes GABAergic inhibition, with its strongest effects on GABAA receptor αaβ2δ receptors [346], causing convulsion. The notorious health problems called absinthism in the past were determined to be not due to thujone but rather caused by alcohol intoxication from wormwood spirit absinthe, as the spirit did not contain a high enough dose of thujone to cause problems [347,348]. A study testing the toxicity of the essential oils of common sage (*S. officinalis*), wormwood (*A. absinthium*), northern white cedar (*Thuja occidentalis*), and tansy (*Tanacetum vulgare*) has shown that toxicity was not solely due to thujones in the oils [349]. These studies indicate that further studies are necessary. There are studies showing that wormwood essential oils improved the cognitive functions of rats with AD (50 mg/kg) [350]. There are also studies showing that thujone has antioxidant and neuroprotective effects, as well as other effects unrelated to dementia [351]. With cautions on the concentrations included in the oils, there are possibilities of utilizing it for AD and other types of dementia.

Enhancing stability is also important, particularly for the purpose of delaying oxidation. For example, the oxidized product of BCP, which is caryophyllene oxide, can be an allergen of moderate strength, with potential to cause skin sensitization [352]. Studies on enhancing the stability of phytochemical compounds while maintaining their pharmacological properties will be important.

### 6.3. The Entourage Effects (Synergistic Effects by Multiple Chemical Compounds)

The entourage effect is best known in studies on the cannabinoid system [353]. Ben-Shabat et al. [354] were the first to call the synergistic effects of multiple chemical compounds ‘the entourage effects’ in their studies on the cannabinoid system. Their study was not on phytochemical compounds but on an endocannabinoid called 2-arachidonoyl-glycerol (2-AG), which showed enhanced binding to cannabinoid receptors 1 and 2 (CB1 and CB2) by endogenous monoglycerides 2-linoleoyl-glycerol (2-LG) and 2-palmitoyl-glycerol (2-PG) [354]. Entourage effects are found in many phytochemical compounds [355,356,357]. For example, a combined use of CBD and BCP produced synergistic analgesic effects [358]. Another well-known example is the combinatory use of curcumin and piperine, improving its bioavailability [359]. Scuteri et al. [360] cited Ribeiro [361] as stating that “the strongest effect of EOs is due to the whole phytocomplex made up of various plant components that need to be present in a precise ratio to exert the so-called entourage effect” (EOs: essential oils) [360,361]. There are multiple factors considered to be involved in producing the synergistic effects, i.e., the entourage effects: (1) enhanced bioavailability by generating a phytocomplex, like in the case of curcumin and piperine [359], (2) additive effects of interfering with different inflammatory pathways, and (3) additive effects of interfering with the same inflammatory pathways [356,362]. Importantly, these synergistic/entourage effects enable reducing the concentration of each phytochemical compound to produce the targeted effects, which can lead to reducing the adverse events, if any. It is important to identify the combinations that work most efficiently and to adjust the concentrations or doses or frequencies depending on the conditions, in addition to the individual effects of each phytochemical compound. For example, factors such as whether it is for prevention or treatment, the specific symptoms of each person, the age, the sex, and other parameters such as the genotype of cytochrome P450 (CYP) [363], which is involved in metabolizing various chemical compounds, should be considered.

### 6.4. Limitations of the Evidence

The reader should be aware that the evidence base for most phytochemicals discussed in this review remains largely preclinical. The majority of mechanistic studies cited were conducted in cell culture systems or rodent models, and the translational validity of such models for complex neurodegenerative diseases like AD is inherently limited. Of the compounds reviewed, those with the most direct and robust clinical evidence in human dementia populations are: EGb 761 (*Ginkgo biloba* extract), with replicated RCT evidence in mild-to-moderate AD and MCI [12,176]; saffron (*Crocus sativus* carotenoids), with two completed RCTs in mild-to-moderate AD [177,178]; curcumin, with clinical trial data in healthy older adults and subjective cognitive decline [9]; and rosmarinic acid, with a completed MCI trial showing improvement on CDR-SB [10]. For all other compounds discussed, clinical translation has not yet been established, and the review’s mechanistic descriptions should be interpreted as hypothesis-generating rather than as evidence of clinical efficacy. Furthermore, many of the clinical studies cited are limited by small sample sizes, short durations, heterogeneous patient populations, and the absence of validated biomarker endpoints. Larger, better-powered trials with standardized formulations and mechanistically relevant endpoints are needed across all compound classes described in this review.

### 6.5. Possibility of Utilizing Plant-Origin Natural Products in Both Prevention and Treatment for ‘Health Span’ in the Longer Life Span

Studies have shown that human life expectancy increased by about 30 years during the 1990s [1], leading to an increase in the number of patients with dementia. How to extend health span or health longevity (the life with good health) is a critical issue in our lives. The importance of exercise and nutrition is often discussed in the literature [364,365]. In addition, phytochemical compounds have a high potential to be used as ‘drugs’ for dementia. A broad range of studies are still needed to learn how to improve the stability of the chemicals, enhance bioavailability, and rationalize the best compounds for specific indications (precision phytochemicals). We can also develop supplements and drugs in addition to using essential oils, and it is important to conduct network machine learning analyses to determine the ‘Hyperplants’ for this goal as well. Medicinal plants have been used since ancient times, but we are still in the early stage of fully employing them based on modern scientific techniques and the evidence from integrated ‘omics’ methodologies with advanced data analyses.

Despite the abundance of preclinical evidence reviewed here, only a small fraction of the phytochemicals with demonstrated activity in cellular and animal models of AD have been evaluated in rigorous human clinical trials. Several interrelated barriers account for this discrepancy. First, many polyphenols, terpenoids, and alkaloids display poor oral bioavailability, undergoing rapid first-pass hepatic metabolism and producing circulating metabolites whose neuroprotective properties may differ substantially from the parent compound. Second, CNS penetration is limited for many hydrophilic or high-molecular-weight phytochemicals, and P-glycoprotein efflux at the blood–brain barrier (BBB) further restricts brain accumulation of some compounds. Third, the chemical heterogeneity of plant-derived preparations, whose composition varies by cultivar, geographic origin, extraction method, and processing conditions, makes dose standardization and reproducibility across trials a significant challenge. Fourth, the typically slow and multifactorial progression of AD demands long-duration, large-cohort trials with validated cognitive and biomarker endpoints that are costly and organizationally complex to maintain for the duration of the trial. Addressing these barriers through improved formulation strategies (as discussed in Section 6.2), rigorous product characterization, and the use of validated surrogate biomarkers in early-phase trials will be essential to translating the preclinical promise of phytochemicals into clinically meaningful therapies.

## Figures and Tables

**Table 1:** Summary of several types of dementia. Based on NIH website (https://www.nia.nih.gov/sites/default/files/understanding-types-dementia_0.pdf, accessed on 22 February 2026) and 2025 Alzheimer’s Association Report [19].

Types of Dementia	Neuropathology	Examples of Symptoms	%
AD	Aβ plaques outside of neurons; tau tangles inside neurons; death of neurons; inflammation and atrophy in brain	Memory loss; wandering; unable to recognize friends and family; impulsive	60–80% of dementia (% differs by reports)
Lewy body dementia	Lewy body protein (abnormal aggregation of α-synuclein in neurons) [21]	Visual hallucinations; visuospatial impairment; insomnia; illogical; difficulty in focusing; problems in motor functions; daytime sleepiness; memory loss	5% of dementia
Frontotemporal dementia	Abnormal amounts or forms of Tau and TDP-43 proteins in frontal and temporal lobes; nerves in frontal and temporal brain die	Impulsive; changes in personality and behavior; shaky hands; problems in balance and walking; difficulties in talking or understanding; during early-stage, memory is not lost	About 3% of patients with dementia older than 65, but 10% of patients with dementia younger than 65
Vascular dementia	Blood clots or injury in the brain disrupting blood flow in the brain	Forgetting current or past events	5–10% of dementia
Hippocampal sclerosis	Accumulation of misfolded protein TDP-43, but in the hippocampus	Memory loss	3–13% of dementia

Note: TDP: transactive response DNA-binding protein; Aβ: amyloid-β.

**Table 2:** Examples of biomarkers for AD.

Biomarker	Changes in Dementia Patients	Other Notes (Sources, etc.)	References
p-Tau181; p-Tau217; p-Tau231	Elevated	Plasma, CSF	[20,26,28]
t-Tau	Elevated	Plasma and CSF	[17]
GFAP	Elevated	Plasma, serum, CSF	[17,28,29]
Aβ42/Aβ40 ratio	Decreased (in Janeiro et al. [17] it is referred to as controversial. In Brickman et al. [26] it is referred to as decreased)	Plasma [26]; CSF [17]	[17,26,28]
Aβ42	Decreased	CSF, plasma	[17]
NfL	Elevated	Plasma [26]; CSF [17]	[17,26,28]
sTREM2	Elevated in CSF but not in plasma	Microglia biomarker	[28]
YKL-40 (CHI3L1)	Elevated along with Aβ accumulation	CSF (plasma YKL-40 does not correlate with Aβ)	[28]
Neurogranin	Decreased	CSF	[17]
Drebrin	Decreased	CSF, plasma	[25]
Small EVs	Increase in EVs that contain the N-terminus-truncated form of Aβ, which degrade less and aggregate more.		[30,31]

Note: Aβ: amyloid-beta; CSF: cerebrospinal fluid; GFAP: glial fibrillary acidic protein; NfL: neurofilament light chain; p-tau: phosphorylated tau; small EVs: small extracellular vesicles; sTREM2: soluble form of the triggering receptor expressed on myeloid cells 2; t-tau: total tau; YKL-40: CHI3L1: Chitinase-3-like protein 1 (a glycoprotein inflammation marker, astrocytic biomarker).

**Table 3:** Examples of FDA-approved drugs for AD. Data was collected mostly from the websites of the Alzheimer’s Association (link above) and the Mayo Clinic.

Name	Target	Route, Frequency	Function/Expected Changes	Adverse Events *
Donanemab (Kisunla™)	Amyloid	IV, every 4 weeks	Removing Aβ; reduction in cognitive decline	Pains, chills, confusion, fainting, fever, nausea, vomiting, etc.
Lecanemab (LEQEMBI)	Amyloid	IV, every 2 weeks	Removing Aβ; reduction in cognitive decline	Pain, chills, confusion, diarrhea, dizziness, drowsiness, etc.
Benzgalantamine (Zunveyl)	AChE	Oral, daily	Suppress breakdown of acetylcholine	Skin reaction, chills, cough, diarrhea, etc.
Donepezil (Aricept)	AChE	Oral or transdermal, daily	Suppress breakdown of acetylcholine	Nausea, severe vomiting, loss of appetite, etc.
Galantamine (Razadyne)	AChE	Oral, daily	Suppress breakdown of acetylcholine	Skin reaction, fever, chills, nausea, diarrhea, etc.
Rivastigmine (Exelon)	AChE	Oral or transdermal, twice daily	Suppress breakdown of acetylcholine	Nausea, vomiting, diarrhea, stomach pain, weight loss, skin rash, etc.
Memantine (Namenda)	Glutamate	Oral, daily	Suppress NMDA receptor	Swelling of face, arms, etc., dizziness, headache, etc.
Donepezil and memantine (Namzaric)	Cholinesterase and glutamate	Oral, daily	Suppress breakdown of acetylcholine and suppress NMDA receptor	Nausea, vomiting, loss of appetite, confusion, dizziness, etc.
Suvorexant (Belsomra)	Orexin receptor	Oral, daily	Inhibit the activity of orexin for treatment of insomnia	Impaired alertness, motor coordination, depression, sleepwalking, sleepiness, drowsiness, etc.
Brexpiprazole (Rexulti)	Anti-psychotic	Oral, daily	Reduce agitation	Weight gain, sleepiness, dizziness, stroke, suicidal thoughts, etc.

Note: IV: intravenous; *: from Mayo Clinic (accessed on 22 February 2026, https://www.mayoclinic.org/drugs-supplements/); AChE: acetylcholinesterase, NMDA: N-methyl-D-aspartate.

**Table 4:** Examples of phytochemicals classified in five phytochemical groups that modulate biological pathways related to dementia and representative plants that contain them.

Groups (Sections)	Key Phytochemicals [49]	Plants [51,52,53]	Biological Pathways (See Each Section for Details)
Flavonoids (Section 4.2)	Kaempferol, catechin, luteolin, cyanidin, apigenin, genistin, quercetin, naringenin, flavokawin C	Chamomile, celery, chrysanthemum flowers, sweet peppers, carrots, onion leaves, broccoli, parsley, nuts, fruits (apples, berries, capers, grapes, onion, tomatoes)	BACE1 inhibition, disruption of Aβ aggregation, tau hyperphosphorylation suppression, inhibition of AChE, SIRT1, PI3K/Akt/GSK-3β, NF-κB/JNK, NF-κB/IKK, TLR4/MyD88/NF-κB, MAPK/JNK, NF-κB/(TNF-α, IL-1β, IL-6, iNOS, and COX-2), MAPK/ERK/CREB, M1/M2 microglial polarization, PPAR-γ/TREM2, Nrf2/ARE, Keap1/Nrf2/HO-1, PI3K/Akt/Nrf2/Keap1, PI3K/Akt/mTOR, AMPK/SIRT1/PGC-1α, SIRT1/iNOS/NF-κB, BDNF/TrkB, Wnt/β-catenin
Alkaloids (Section 4.3)	Morphine, cocaine, caffeine, solanin, nicotine, spermine, capsaicin, berberine, galantamine, harmine	Jasmine, barberry, turmeric, goldenseal, Chinese cork tree, toothed clubmoss, coffee bean	MAO inhibition, NF-κB/MAPK, NF-κB/MAPK/STAT3, NF-κB/IKK, TLR4/NF κB, JNK-p38MAPK, MAPK/ERK, PI3K/Akt/ERK, mTOR/p70S6K, PI3K/beclin-1, α7nAChR/Nrf2/Keap1, Akt/GSK-3β/Tau, PI3K/Akt/GSK-3β, IGFR-mediated JNK-Akt, NLRP3-dependent inflammatory signaling, inhibition of AChE, Aβ aggregation inhibition, tau hyperphosphorylation suppression
Terpenoids (Section 4.4)	Menthol, artemisinin, phytol, tetraterpenes, oleanolic acid, polyterpenoids, linalool, limonene, β-caryophyllene, ginkgolides	Lavender, rosemary, frankincense, lemon balm, peppermint, sandalwood, ylang-ylang, chamomile, eucalyptus, clary sage, sweet orange, jasmine, vetiver, bergamot, geranium, marjoram, neroli, patchouli, tea tree, cinnamon, ginger, lemongrass, myrrh, juniper berry, angelica root, inula viscosa, ginkgo, saffron	Aβ aggregation inhibition, tau hyperphosphorylation suppression, inhibition of AChE, NF-κB/(TNF-α, IL-1β, and IL-6), NF-κB/IκBα/p65, NF-κB/IKKβ, PI3K/Akt/GSK-3β, Wnt/GSK-3β/β-catenin, Nrf2/HO-1, PI3K/Akt/Keap1/Nrf2, AMPK/Keap1/Nrf2, PI3K/Akt/mTOR, MAPK/ERK, PI3K/Akt/GSK-3β, BDNF/TrkB, BDNF/CREB
Phenolic Components (Section 4.5)	Cinnamic acid, syringic acid, vanillic acid, ferulic acid, L-DOPA, p-hydroxybenzoic acid, rosmarinic acid, p-coumaric acid, eugenol, thymol, carvacrol, curcumin	Basil, thyme, cinnamon, clove, oregano, parsley, turmeric, rosemary, lemon balm, grapes, cereal seeds (wheat, oats, rye, and barley), whole grain, spinach, artichokes, coffee, blueberries, sandalwood	Nrf2/HO-1, NF-κb/Nrf2/Bax, NF-κB/MAPK, MAPK/ERK, SIRT1/NF-κB, PPARγ/NF-κB, ERK/JNK/p38, GSK3β/PTEN/PI3K/Akt, GSK3β-Nrf2, SIRT3/SOD2, COX-2, TNF-α and IL-1β inflammation pathways, Aβ aggregation and tau phosphorylation signaling, AMPK/PGC-1α/Sirt3, inhibition of AChE
Phytosterols (Section 4.6)	Ergosterol, brassicasterol, β-sitosterol, β-sitostanol, campesterol, campestanol, stigmasterol, resveratrol, fucosterol, 24(S)-saringosterol	Grapes, peanuts, blueberries, vegetable oils, nuts, whole grains	BCAE1/APP, AMPK/NF-κB, AMPK/NLRP3, PI3K/GSK-3β, inhibition of AChE, PI3K/Akt/GSK-3β, ERK/CREB, LXR/ABCA1, LXR/ABCG1, Aβ aggregation inhibition

Note: α7nAChR: α7 nicotinic acetylcholine receptor, ABCA1: ATP-Binding Cassette Transporter A1, ABCG1: ATP-Binding Cassette Transporter G1, APP: amyloid precursor protein, Akt: Ak strain transforming, AMPK: AMP-activated protein kinase, ARE: Antioxidant response element, Bax: Bcl-2-associated X protein, Bcl-2: B-cell lymphoma 2, BCAE1: Beta-site amyloid cleaving enzyme 1, COX-2: Cyclooxygenase-2, BDNF: Brain-derived neurotrophic factor, CREB: cAMP response element-binding protein, L-DOPA: L-3,4-dihydroxyphenylalanine, ERK: Extracellular signal-regulated kinase, GSK3β: Glycogen synthase kinase-3β, HO-1: Heme oxygenase-1, IGFR: insulin-like growth factor receptor, IκB: Inhibitor of nuclear factor kappa Bα, IKK: Inhibitor of nuclear factor-κB (IκB) kinase, IL-1β: Interleukin-1β, IL-6: Interleukin-6, iNOS: Inducible nitric oxide synthase, JNK: c-Jun NH2-terminal kinase, Keap1: Kelch-like enoyl-CoA hydratase-associated protein, LXR: Liver X receptor, MAO: Monoamine oxidases, MAPK: Mitogen-activated protein kinase, mTOR: Mechanistic target of rapamycin, NF-κB: Nuclear Factor kappa-light-chain-enhancer of activated B cells, NLRP3: Nucleotide-binding oligomerization domain-like receptor family pyrin domain containing 3, Nrf2: Nuclear factor erythroid 2-related factor 2, p38: A mitogen-activated protein, p70S6K: p70 Ribosomal S6 Kinase, PGC-1α: Peroxisome proliferator-activated receptor gamma coactivator 1-α, PI3K: Phosphoinositide 3-kinase, PPARγ: Peroxisome proliferator-activated receptor γ, PTEN: Phosphatase and tensin homolog, SIRT1: Sirtuin (silent mating type information regulation 2 homolog) 1, SOD2: Mitochondrial superoxide dismutase 2, STAT3: Signal transducer and activator of transcription 3, TLR4: Toll-like receptor 4, TNF-α: Tumor Necrosis Factor α, TREM2: Triggering receptor expressed on myeloid cell 2, TrkB: Tropomyosin receptor kinase B, Wnt: Wingless-related integration site.

**Table 5:** Summary of the examples of phytochemical compounds shown in the main text of Section 3.

Groups (Location in the Text)	Inhibition/Suppression of Aβ Aggregation	Inhibition/Suppression of Tau Aggregation	Inhibition/Suppression of Neuro-Inflammation	Anti-Oxidation	Modulation of Key Signal Transduction Pathways	Adult Neurogenesis	Inhibition/Suppression of AChE, BChE
Flavonoids (Section 4.2)	EGCG, Epigallocatechin, fisetin, kaempferol, luteolin, myricetin, naringenin quercetin, rutin, taxifolin	EGCG, fisetin kaempferol, luteolin, quercetin, myricetin rutin	apigenin, kaempferol, luteolin, naringenin, quercetin	apigenin, EGCG, genistein, quercetin	cyanidin-3-*O*-glucoside (-)epicatechin, 7,8-dihydroxyflavone fisetin, kaempferol, luteolin, myricetin, naringenin quercetin	anthocyanins, flavanols, quercetin	chrysin, EGCG, kaempferol, luteolin, quercetin
Alkaloids (Section 4.3)	berbamine, berberine, harmine, P4B, sinomenine	allocryptopine, berberine, dendrobium nobile lindl. alkaloid, harmine, kopoffines, tetrahydropalmatine, tetrahydroberberine	allocryptopine, harmine, isoliensinine, liensinine, neferine, tetrandrine, tetrahydropalmatine, tetrahydroberberine N-oxide, vinpocetine	berberine, harman, harmine, sinomenine	Dendrobium nobile lindl. alkaloid, tetrandrine, isoliensinine, berberin, sinomenine, vinpocetine	berberine, huperzine A, harmine, galantamine, vinpocetine	galantamine, harmine derivatives, berberine
Terpenoids (Section 4.4)	bacosides, β-caryophyllene crocin, cornel iridoid glycoside, cryptotanshi-none, ginkgolide B, withanolides	asiaticosides, bacosides, cornel iridoid glycoside, cryptotanshi-none, ginsenoside Rg1, ursolic acid, withanolides	bacosides, bilobalide, β-caryophyllene, 1,8-cineole, cornel iridoid glycoside, cryptotanshi-none, ginkgolide B, ginsenoside Rg1, linalool, tanshinone IIA, ursolic acid, withanolides	asiaticosides, bacosides, bilobalide, crocin, ginsenosides, withaferin A	asiaticosides, bacosides, crocin, cornel iridoid glycoside, cryptotanshi-none, ginkgolides, ginsenoside Rg1, safranal, withanolides	asiaticosides, bacosides, ginsenoside Rg1	1,8-cineole, citral, citronellal, ginsenosides, linalool, α-pinene safranal, ursolic acid
Phenolic components (Section 4.5)	Caffeic acid, ferulic acid, forsythoside A, rosmarinic acids	ferulic acid	Gallic acid, phloretin, protocatechuic acid resveratrol, rosmarinic acid, sesamol, tetrahydrocurcumin, trilobatin	phenethyl ester 4-*O*-glucoside, sesamol	Caffeic acid phenethyl ester 4-*O*-glucoside, honokiol, urolithin, resveratrol, phloretin, resveratrol, tetrahydrocurcumin, trilobatin, gallic acid	Propofol, vanillic acid, caffeic acid alkyl esters, rosmarinic acid, gallic acid	Caffeic acid, ferulic acid, rosmarinic acid, gallic acid, vanillic acid
Phytosterols (Section 4.6)	fucosterol, β-sitosterol, stigmasterol		campesterol, β-sitosterol, stigmasterol	β-sitosterol, stigmasterol	β-sitosterol, stigmasterol, fucosterol, 24(S)-saringosterol		fucosterol, β-sitosterol, stigmasterol

**Table 6:** Examples of observations of changes after hand massage using essential oils (unpublished data). Age > 80. One male patient and all others were female patients.

Patterns	Client	Before	After
Pattern 1 (mood)	Dementia patient	The patient is usually agitated, upset, or grumpy	About 6 min after the massage started, the patient calmed down with a calm look
Pattern 2 (mood)	Dementia patient	No description about before the hand massage	Started to smile, and the face lit up
Pattern 3 (talking)	Dementia patient	Patient did not talk about family at the facility; Patient had negative self-esteem	Started to talk about their family; Started to talk with the aromatherapist; Repeatedly talked about the same content; Started to talk, thank, and smile
Pattern 4 (falling asleep)	Dementia patient	A patient who talks usually calmly or quietly	Around the time the massage of one hand is over and switching to the other hand (about 12 min for each hand), the patient started to fall asleep
Pattern 5 (emotional)	Dementia patient	Has emotional instability, which gradually became worse as the symptoms of dementia progressed. Many patients showed an increase in anxiety late in the afternoon.	Suddenly became overcome with emotion and started to cry when the hand massage started, then the mood became uplifted, starting to happily say it feels very nice, returning to her/his room calmly
Pattern 6 (curiosity)	Healthy 97-year-old senior	Previously worked in a company related to drug development	Showed a strong interest in the name of the essential oil and asked for its name

**Table 7:** Examples of the essential oils or the plants and their major constituents with effects on factors associated with dementia.

Effects	Essential Oil	Chemical Constituents Proposed to Have Effects on Dementia	References
Anti-Aβ	Ashwagandha * (*Withania somnifera*)	Withanolides ^#4.4^	[47]
	Asian ginseng * (*Panax ginseng*)	Ginsenoside ^#4.4^	[47,290]
	Aleppo pine * (*Pinus halepensis*)	β-Caryophyllene ^#4.4^, α-pinene ^#4.4^ and myrcene	[291,292]
	Cinnamon (*Cinnamomum zeylanicum*)	Linalool ^#4.4^, (E)-cinnamaldehyde and (E)-cinnamyl acetate, eugenol [293]; italicene [294]	[293,294]
	Coriander (*Coriandrum sativum*)	Linalool ^#4.4^	[295]
	Ginkgo * (*Ginkgo biloba*)	Ginkgolides ^#4.4^ (A,B,C,J), bilobalide ^#4.4^	[47]
	Indian pennywort (or gotu kola, Asian pennywort, Ji Xue Cao, *Centella asiatica*)	Asiaticoside ^#4.4^, madecassoside, asiatic acid, madecassic acid; isochlorogenic acid A, 1,5-dicaffeoylquinic acid	[47,283,296,297]
	Lavender (*Lavandula angustifolia*)	Linalool ^#4.4^, linalyl acetate, β-caryophyllene **** ^#4.4^	[295]
	Lemon balm (*Melissa officinalis*)	2,2-Dimethoxybutane, 3′,4′,5,7-tetrahydroxyflavone, 3′-O-β-D-glucuronopyranoside, γ-O-β-D-glucopyranoside and 2,3,19,23-tetrahydroxy-12-ursen-28-oic acid-23-sulfate, 28-O-β-D-glucopyranosyl ester, sajerinic acid [298]; solely through rosmarinic acid ^#4.5^ [47]	[47,298]
	Rosemary * (*Rosmarinus officinalis*)	Oleanolic acid	[294]
	Saffron * (*Crocus sativus*)	Crocin ^#4.4^, safranal ^#4.4^	[47]
	Sage ** (*Salvia officinalis*)	Pinocembrin [294]; rosmarinic acid ^#4.5^, carnosic acid	[47,294]
	Turmeric (*Curcuma longa*)	Curcumin ^#4.5^, demethoxycurcumin, bisdemethoxycurcumin	[47]
	Waterhyssop * (*Bacopa monnieri*)	Bacosides A ^#4.4^, bacosides B ^#4.4^	[47]
	Sweet wormwood (*Artemisia annua*)	Artemisinin	[299,300]
Anti-tau	Cinnamon (*Cinnamomum zeylanicum*)	Chamigrene, italicene	[294]
	Rosemary (*Rosmarinus officinalis*)	Luteolin ^#4.2^	[294]
	Sage ** (*Salvia officinalis*)	Myricetin ^#4.2^	[294]
	Saffron * (*Crocus sativus*)	Crocin ^#4.4^, safranal ^#4.4^	[47]
	Sweet wormwood (*Artemisia annua*)	Artemisinin	[299,300]
Anti-α-Synuclein	Citron * (*Citrus medica*)	Linalool ^#4.4^	[293]
	Sage ** (*Salvia officinalis*)	1,8-Cineole (eucalyptol) ^#4.4^	[293]
	Thyme (*Thymus vulgaris*)		[292]
Anti-AChE	Ashwagandha * (*Withania somnifera*)	Withanolides ^#4.4^	[47]
	Cinnamon (*Cinnamomum zeylanicum*)	Linalool ^#4.4^, (E)-cinnamaldehyde and (E)-cinnamyl acetate, eugenol	[293]
	Clove (*Syzygium aromaticum*)		[301]
	False daisy * (*Eclipta prostrata*)	Wedelolactone	[302,303]
	Garlic (*Allium sativum*) *	Allicin [304]	[304,305]
	Greater galangal * (*Alpinia galanga*)	α-Humulene [306]	[306,307,308]
	Lemon balm (*Melissa officinalis*)	2,2-Dimethoxybutane, 3′,4′,5,7-tetrahydroxyflavone, 3′-O-β-D-glucuronopyranoside, γ-O-β-D-glucopyranoside and 2,3,19,23-tetrahydroxy-12-ursen-28-oic acid-23-sulfate, 28-O-β-D-glucopyranosyl ester, sajerinic acid [298]	[47,309]
	Myrtle (*Myrtus cummunis*)		[310]
	Oregano (*Origanum vulgare*)	Thymol, carvacrol	[292]
	Black pine (*Pinus nigra*)		[311]
	*Pistacia khinjuk* *		[305]
	Rosemary (*Rosmarinus officinalis*)	1,8-Cineole (eucalyptol) ^#4.4^	[292,294]
	*Tetraclinis articulata* *		[312]
	True cinnamon tree (*Cinnamomum verum*)	(E)-Cinnamaldehyde [310]	[310]
Anti-butyrylcholinesterase	False daisy * (*Eclipta prostrata*)	Wedelolactone	[302,303]
	Myrtle (*Myrtus cummunis*)		[310]
	*Pinus nigra*		[311]
	True cinnamon tree (*Cinnamomum verum*)	(E)-Cinnamaldehyde [310]	[310]
Anti-beta-Secretase 1	Lemon balm (*Melissa officinalis*)	Triethyl citrate, 3′,4′,5,7-tetrahydroxyflavone, 3′-O-β-D-glucuronopyranoside, γ-O-β-D-glucopyranoside and 2,3,19,23-tetrahydroxy-12-ursen-28-oic acid-23-sulfate, 28-O-β-D-glucopyranosyl ester, sajerinic acid	[298]
	True cinnamon tree (*Cinnamomum verum*)	(E)-Cinnamaldehyde [310]	[310]
	Turmeric (*Curcuma longa*)	α-Turmerone, β-turmerone and turmerone	[313]
Anti-neuroinflammation	Bergamot *** (Citrus bergamia)	Limonene, linalyl acetate ****, Linalool **** ^#4.4^	[292]
	Clove (*Syzygium aromaticum*)	Eugenol	[292]
	Ginger (*Zingiber officinale*)	10-gingerol, 6-shogaol	[292]
	Lavender (*Lavandula angustifolia*)	Linalool ^#4.4^, linalyl acetate	[292]
	Peppermint (*Mentha piperita*)	Menthol, 1,8-Cineole ****	[292]
	*Satureja khuzistanica* *	Carvacrol	[292]
	Tea tree (*Melaleuca alternifolia*)	Terpinene-4-ol, 1,8-Cineole **** ^#4.4^	[292]
	Thyme (*Thymus vulgaris*)		[292]
	Sweet wormwood (*Artemisia annua*)	Artemisinin	[299,300]

*: Not commonly used as essential oils but on the market as supplements; **: Contains thujone, which is safe for culinary use but needs caution when used in higher doses as tea, medicines, supplements, and essential oils. See Section 6.2; ***: Contains bergapten, which is a furocoumarin. See Section 6.2; ****: Chemical constituents usually listed to be contained in the essential oils of these plants by the Japan Aromacoordinator Association (JAA) and found to have effects on dementia but not specifically referred to in the paper cited; #: Chemical compounds with description in Section 4 with the section number. For example, #4.4 indicates it is described in Section 4.4.

**Table 8:** Phytochemical compounds with a high correlation to drugs for AD (from Rita et al. [334]).

Phytochemical Compound	Correlation %
Quercetin	78
Genistein	75.5
Luteolin	73.9
Palmitoleate	69.5
Stearic acid	67.5
Apigenin	67.4
Epicatechin	66.4
Kaempferol	65.1
Squalene	63.7
Daidzein	62.1

## Data Availability

The original contributions presented in this study are included in the article.

## Abbreviations

The following abbreviations are used in this manuscript:

α7nAChR	α7 nicotinic acetylcholine receptor
ABCA1	ATP-binding cassette transporter A1
ABCG1	ATP-binding cassette subfamily G member 1
Aβ	Amyloid-β
AChE	Acetylcholinesterase
AD	Alzheimer’s disease
ADAS-cog	Alzheimer’s disease assessment scale
ADRD	Alzheimer’s disease and related dementias
2-AG	2-Arachidonoyl-glycerol
Akt	Ak strain transforming
AMPK	AMP-activated protein kinase
ApoE	Apolipoprotein E
APP	Amyloid precursor protein
ARE	Antioxidant response element
ATP	Adenosine triphosphate
BACE1	Beta-site amyloid precursor protein cleaving enzyme 1
Bax	Bcl-2-associated X protein
BBB	Blood–brain barrier
Bcl-2	B-cell lymphoma 2
BCAE1	Beta-site amyloid cleaving enzyme 1
BDNF	Brain-derived neurotrophic factor
BChE	Butyrilcholinesterase
CB1, CB2	Cannabinoid receptor 1, cannabinoid receptor 2
CBD	Cannabidiol
CDC	U.S. Centers for Disease Control and Prevention
CDK5	Cyclin-dependent kinase 5
CDR	Clinical Dementia Rating
CDR-SB	Clinical Dementia Rating-Sum of the Boxes
ChAT	Choline acetyltransferase
CHI3L1	Chitinase-3-like protein 1
CMAI	Cohen-Mansfield Agitation Inventory
CNPI	Chinese version of Neuropsychiatric Inventory
CNS	Central nervous system
COX-2	Cyclooxygenase-2
CREB	Cyclic-AMP response element-binding protein
CSF	Cerebrospinal fluid
CYP	Cytochrome P450
DCX	Doublecortin
DSM-5	The Diagnostic and Statistical Manual of Mental Disorders, 5th Edition by the American Psychiatric Association
DYRK1A	Dual specificity tyrosine-phosphorylation-regulated kinase
EGCG	Epigallocatechin-3-gallate
ERK	Extracellular signal-regulated kinase
FDA	The U.S. Food and Drug Administration
FITC	Fluorescein isothiocyanate
FXR	Farnesoid X receptor
EV	Extracellular vesicle
FA-97	Caffeic acid phenethyl ester 4-*O*-glucoside
FAST	Functional Assessment Staging of Alzheimer’s disease
GBSS-J	Japanese version of Gottfries, Braine, Steen Scale
GC-MS	Gas chromatography-mass spectrometry
GFAP	Glial fibrillary acidic protein
GGAE	Allocryptopine, tetrahydropalmatine, and tetrahydroberberine N-oxide rich alkaloid extract from *Glaucium grandiflorum*
GSK3β	Glycogen synthase kinase-3 beta
hAChE	Human acetylcholinesterase
HDS-R	Hasegawa’s dementia scale
HO-1	Heme oxygenase-1
ICD-11	International Classification of Diseases 11th Revision
IDE IGFR	Insulin-degrading enzyme Insulin-like growth factor receptor
IκBα	Inhibitor of nuclear factor kappa B alpha
IKK	Inhibitor of nuclear factor-κB (IκB) kinase
IL-1β	Interleukin-1 beta
IL-6	Interleukin-6
iNOS	Inducible nitric oxide synthase
IV	Intravenous
JNK	c-Jun NH2-terminal kinase
Keap1	Kelch-like enoyl-CoA hydratase-associated protein
Ki-67	Kiel-67 (a nuclear protein)
L-DOPA	L-3,4-dihydroxyphenylalanine
LTP	Long-term potentiation
LXRα and LXRβ	Liver X receptors
MAO	Monoamine oxidases
MCI	Mild cognitive impairment
MAP2	Microtubule-associated protein 2
MAPK	Mitogen-activated protein kinase
mTOR	Mammalian target of rapamycin
NF-κB	Nuclear Factor kappa-light-chain-enhancer of activated B cells
NFL	Neurofilament light chain
NIA-AA	National Institute of Aging and the Alzheimer’s Association
NLRP3	Nucleotide-binding oligomerization domain-like receptor family pyrin domain containing 3
NMDA	N-methyl-D-aspartate
NPCs	Neural progenitor cells
NSPCs	Neural stem and progenitor cells
NQO1	NAD(P)H:quinone oxidoreductase 1
Nrf2	Nuclear factor erythroid 2-related factor 2
OX1R, OX2R	Orexin receptor 1, orexin receptor 2
p38	A mitogen-activated protein
p70S6K	p70 Ribosomal S6 Kinase
PC-12	Pheochromocytoma-derived cell line
PCC	Posterior cingulate cortex
2-PG	2-Palmitoyl-glycerol
PGC-1α	Peroxisome proliferator-activated receptor gamma coactivator 1-alpha
PI3K	Phosphoinositide 3-kinase
PPARγ	Peroxisome proliferator-activated receptor gamma
PSD-95	Postsynaptic density protein 95
PS1	Presenilin-1
p-tau	Phosphorylated tau
PTEN	Phosphatase and tensin homolog
ROS	Reactive oxygen species
SCFA	Short-chain fatty acid
SCOP	Scopolamine
SH-SY5Y cells	A thrice-subcloned cell line derived from the SK-N-SH neuroblastoma cell line
SIRT1	Sirtuin (silent mating type information regulation 2 homolog) 1
SIRT3, Sirt3	Sirtuin (silent mating type information regulation 2 homolog) 3
SLN	Solid lipid nanoparticles
SOD2	Mitochondrial superoxide dismutase 2
STAT3	Signal transducer and activator of transcription 3
sTREM	Soluble form of the triggering receptor expressed on myeloid cells 2
SYN	The gene that encodes the alpha-synuclein protein
TDP	Transactive response DNA-binding protein
TFEB	Transcription factor EB
TGR5	Takeda G protein-coupled receptor 5
TLR4	Toll-like receptor 4
TNF-α	Tumor Necrosis Factor alpha
TREM2	Triggering receptor expressed on myeloid cell 2
TrkB	Tropomyosin receptor kinase B
T-tau	Total tau
WAIS-III	Weschler Adult Intelligence Scale (ver.3)
WHO	World Health Organization
Wnt	Wingless-related integration site
YKL-40	Chitinase-3-like protein 1 (CHI3L1)
3 × Tg AD	Triple-transgenic mouse model of Alzheimer’s disease
